# Profiles of research waste in randomized clinical trials comparing de-escalation and escalation strategies for breast cancer treatment

**DOI:** 10.3389/fonc.2026.1935566

**Published:** 2026-09-24

**Authors:** Yi-Hui Tang, Wei-Jie Lin, Ze-Bin Li, Ju-Jiang Guo, He-Hui Mao

**Affiliations:** Department of Breast Surgery, Women and Children’s Hospital, School of Medicine, Xiamen University, Xiamen, China

**Keywords:** breast cancer, de-escalation studies, escalation studies, premature discontinuation, research waste

## Abstract

**Background:**

Cancer clinical trials produce a substantial amount of research waste. The relationship between different study types, such as de-escalation and escalation studies, and research waste has not been studied.

**Methods:**

This cross-sectional study reviewed phase 2, 3 and 4 randomized clinical trials of breast cancer therapy registered at ClinicalTrials.gov between 2011-2021. Research waste was defined as the presence of one or more of the following three features: nonpublication, inadequate reporting, and design limitations. Studies were categorized as de-escalation, escalation, or other treatment optimization studies, based on the interventions in the experimental and control groups.

**Results:**

In total, 240 studies were included, including 15 de-escalation studies, 139 escalation studies, and 86 other treatment-optimization studies. De-escalation studies were more likely to be non-pharmacological (26.7%), national (86.7%), or non-industry-funded (73.3%). Escalation studies were more likely to have a palliative intent (71.2%) and a double-blind design (42.4%). Heterogeneity among the three study types was observed across the three components of research waste as well as study discontinuation. Overall, 113 of the 240 studies (47.1%) demonstrated research waste. After adjusting for study characteristics such as design and discontinuation, escalation studies were independently associated with an increased risk of research waste compared to de-escalation studies (odds ratio, 4.337; 95% confidence interval, 1.165–18.567; P = 0.028).

**Conclusion:**

A substantial burden of research waste was observed among phase 2 to 4 breast cancer treatment studies. The burden of research waste varied across study types, highlighting the need for tailored strategies to mitigate waste. Given the limited number of de-escalation studies, these exploratory findings require further validation.

## Introduction

Clinical trials are designed to determine the efficacy and safety of therapeutic interventions by comparing treatment effects (benefits or harms) at prespecified endpoints with those of control groups (1). These trials provided a basis for determining whether an intervention should be recommended. As a substantial proportion of human clinical trials, cancer clinical trials are essential for improving global cancer control (2). However, they are becoming increasingly complex and costly, leading to declining efficiency and quality, and exposing a substantial burden of research waste (3). In this era of rapidly evolving therapeutic landscapes, minimizing research waste is ethically imperative to facilitate the global translation of study findings (4, 5).

Although multiple studies have examined research waste in cancer clinical trials (particularly randomized controlled trials [RCTs]) (6–8), certain limitations cannot be ignored. A notable shortcoming is that these studies have primarily evaluated the external factors of the trials, such as funding, blinding, and recruitment, in relation to research waste, while overlooking their intrinsic characteristics. For example, studies categorized as de-escalation or escalation based on their objectives typically display distinct characteristics. Escalation studies are frequently initiated by pharmaceutical companies, whereas de-escalation studies often require academic efforts and public funding. The relationship between the study type and research waste has not yet been explored.

We hypothesized that de-escalation studies would be more prone to research waste than escalation studies. Therefore, we conducted an observational study to evaluate the burden of research waste in phase 2 to 4 breast cancer RCTs and its association with study type (escalation vs. de-escalation).

## Materials and methods

### Study design

This was a cross-sectional study of RCTs registered in the ClinicalTrials.gov database. We chose this database because ClinicalTrials.gov is the most comprehensive and publicly accessible clinical trial registry and provides updated information on clinical trials. Because this study involved a review of publicly available data, the Institutional Review Board of our center waived the requirements for ethical approval and informed consent. This study adhered to the Strengthening of the Reporting of Observational Studies in Epidemiology (STROBE) guidelines.

### Search strategy

To identify the eligible studies, the following filters were utilized on a single day (November 11, 2025): “(breast cancer) OR (breast carcinoma) OR (breast neoplasm) OR (mammary neoplasm) OR (mammary cancer) OR (ductal carcinoma *in situ*)” as the “Condition/disease,” “interventional” as the “Study Type,” and “phase 2, 3 and 4” as the “Study Phase.” We limited the eligible studies to those registered between January 1, 2011, and December 31, 2021, with a study completion date no later than December 31, 2022. We chose this completion date because a 36-month interval allows most studies to publish their findings after completion.

### Eligibility criteria

The search results were downloaded and converted into an Excel file. The following studies were initially excluded based on the relevant registry fields: non-randomized (allocation); single-group (Intervention Model); male (sex); diagnostic, prevention, supportive care, health services research, basic science, or other (Primary Purpose); phase 1/2 (phase); and dietary supplements (interventions). The following studies were further excluded: (1) not related to or not limited to breast cancer, (2) not involving anti-cancer therapy, (3) generic drug studies, (4) non-randomized or phase 1 studies, (5) pooled or extension analysis of other studies, and (6) non-English publications. Phase 1 studies were excluded as their primary aim is preliminary efficacy/safety evaluation, with no routine plan for publication. Studies registered as “Not Applicable” for phase were excluded, as the vast majority investigate behavioral interventions or quality improvement initiatives rather than anti-cancer therapies. Studies examining non-conventional anticancer treatments (e.g., metformin) were also excluded. Study screening was conducted by one investigator and independently reviewed by a second investigator. Any discrepancies were resolved through discussion.

### Data collection

Data were extracted from the results exported by one investigator, recorded in a preformatted document, and shared with the remaining researchers. The extracted information includes the following:

1. Study: NCT number, study title, phase, start date, completion date, and sponsor;
2. Population: sample size (target and actual), recruitment, and tumor subtype
3. Design: phase, masking, and study type
4. Intervention: arm (experimental or control), intent, and intervention type.

Most information (e.g., sample size and masking) could be directly extracted from the exported data, whereas some information required manual summarization by the investigator as detailed below: The summarized information was reviewed by another investigator and any discrepancies were resolved through discussion. Recruitment was categorized as international or national according to the participating centers provided in the “locations” field. Tumor subtypes were categorized according to the “eligibility criteria” as hormone receptor (HR)-positive/human epidermal growth factor receptor 2 (HER2)-negative, HER2-positive (irrespective of the HR status), triple-negative, other (not classifiable into the aforementioned categories), or unspecified. Treatment intent was categorized as curative or palliative according to the “detailed description” and “eligibility criteria.” Intervention type was categorized as pharmacological or non-pharmacological according to the “interventions” field, and pharmacological interventions were further classified as cytotoxic, antibody, small molecule inhibitor, endocrine, or other, according to the specific intervention.

Studies were categorized as de-escalation, escalation, or other treatment optimization studies, according to their arms. De-escalation and escalation studies were defined based on modifications to the standard treatment intensity. De-escalation studies involve a reduction in dose density, dose intensity, or cumulative dose, adoption of intermittent schedules, shortening of treatment duration, or removal of components, segments, compounds, or modalities with the goal of maintaining efficacy and safety (9). In contrast, escalation studies involve increasing treatment intensity or adding components to enhance therapeutic outcomes. Other treatment optimization studies include investigations of different dose schedules, sequences, routes of administration, and treatment modalities (10). Notably, studies conducted in a curative setting that omitted chemotherapy in favor of endocrine therapy for HR-positive breast cancer were classified as de-escalation studies (11).

### Outcome measurements

The primary outcome of this study was the burden of research waste among the RCTs of breast cancer therapy. As reported by Chapman et al., a study was considered wasted if it exhibits at least one indicator of potential research waste (i.e., nonpublication, inadequate reporting, or design limitations) (12). Under this framework, all unpublished studies were considered wasted by default, and published studies with either inadequate reporting or design limitations are likewise considered wasted.

First, a study was considered published if a full-text manuscript was identified in a peer-reviewed journal. The publication status was determined by searching PubMed, Embase, and Google Scholar using combinations of multiple keywords, including NCT number, intervention, and investigator surnames. If multiple publications were identified, only the publication that first reported the primary endpoint was used for the assessment. If no results were identified, searches were sequentially repeated using each of the aforementioned keywords with cross-verification against additional registry information (e.g., study population, sample size, and recruitment period). The searches were conducted between December 10 and 15, 2025.

Second, the quality of reporting in each published trial was assessed according to the Consolidated Standards of Reporting Trials (CONSORT) 2010 checklist (**Supplementary Table 1**). The CONSORT checklist contains a list of essential items that should be included in RCT reports. Following Chapman et al., we applied the following modifications: studies in journals with title length limits (i.e., *The New England Journal of Medicine*) or unstructured abstracts were exempted from items 1a and 1b; non-comparative studies were exempted from effect estimate items 17a and 17b; studies without binary endpoints were exempted from item 17b; and certain conditional items (e.g., 3b, 6b, and 7b) were not assessed. Studies with ≤25% of applicable items (9 items for pharmacological studies, 10 for non-pharmacological studies) unreported were considered adequately reported. Notably, despite the release of CONSORT 2025, the 2010 version was used for evaluation, as nearly all included studies were conducted under this framework.

Third, a new approach was adopted to assess the design limitations. Unlike Chapman et al., the failure to cite relevant systematic reviews was not considered a part of the design limitations owing to its one-sidedness. We used the Cochrane Collaboration’s Bias 2.0 tool to evaluate the Risk of Bias (13), and studies judged as high-risk were considered to have design limitations. Compared to version 1.0, 2.0 version provides a more systematic and standardized assessment with increased reliability. Studies rated as having “some concerns” were not regarded as having design limitations, as these were mostly due to insufficient reporting rather than true design limitations.

Secondary outcomes included the incidence of each component of research waste and study discontinuation. Based on the recruitment status in the registry, studies were categorized as completed or discontinued, and specific reasons for discontinuation were obtained from the registry or publications. Specifically, poor recruitment, organizational or strategic reasons, and limited resources were regarded as preventable causes (14). Similarly, primary and secondary outcomes were independently assessed by two investigators. Discrepancies were resolved through discussion. Agreement between two investigators was assessed using Cohen’s kappa (κ). Inter-rater agreement was excellent across all domains, with κ values of 0.98, 0.89, and 0.94 for nonpublication, inadequate reporting, and design limitations, respectively.

### Statistical analysis

All statistical analyses were conducted using R version 4.4.0 for Windows (R Foundation for Statistical Computing, Vienna, Austria). Categorical variables were compared using the chi-square test, Fisher’s exact test, or Mann–Whitney U test, as appropriate. Univariate and multivariate logistic regression analyses were performed and odds ratios (ORs) and their corresponding 95% confidence intervals (CIs) were calculated. Variables with a P value <0.2 in the univariate analysis were included in the multivariate analysis. To analyze the association between study type and research waste, we selected Firth penalized logistic regression, as it produces more reliable estimates than standard logistic regression when applied to sparse, unbalanced data. Statistical significance was set at P < 0.05. significant.

## Results

### Study characteristics

Our search of ClinicalTrials.gov yielded 1391 clinical trials on breast cancer. After screening against the eligibility criteria, 240 studies involving 72,529 participants (range, 2–4804; median, 163) were included in our analysis (**Figure 1A**, **Supplementary Table 2**). Among these, 142 (59.2%) were labeled as phase 2, 93 (38.8%) as phases 2/3 or 3, and 5 (2.1%) as phase 4. The majority of studies (95.0%, 228 of 240) investigated pharmacological interventions, and the treatment intention was palliative in 62.5% (150 of 240). The median target sample size was 200 (range, 30–3806), and half of the studies recruited participants from multiple nations. Nearly 70% of the studies (167 of 240) received funding from the industry (**Table 1**).

**Figure 1:**
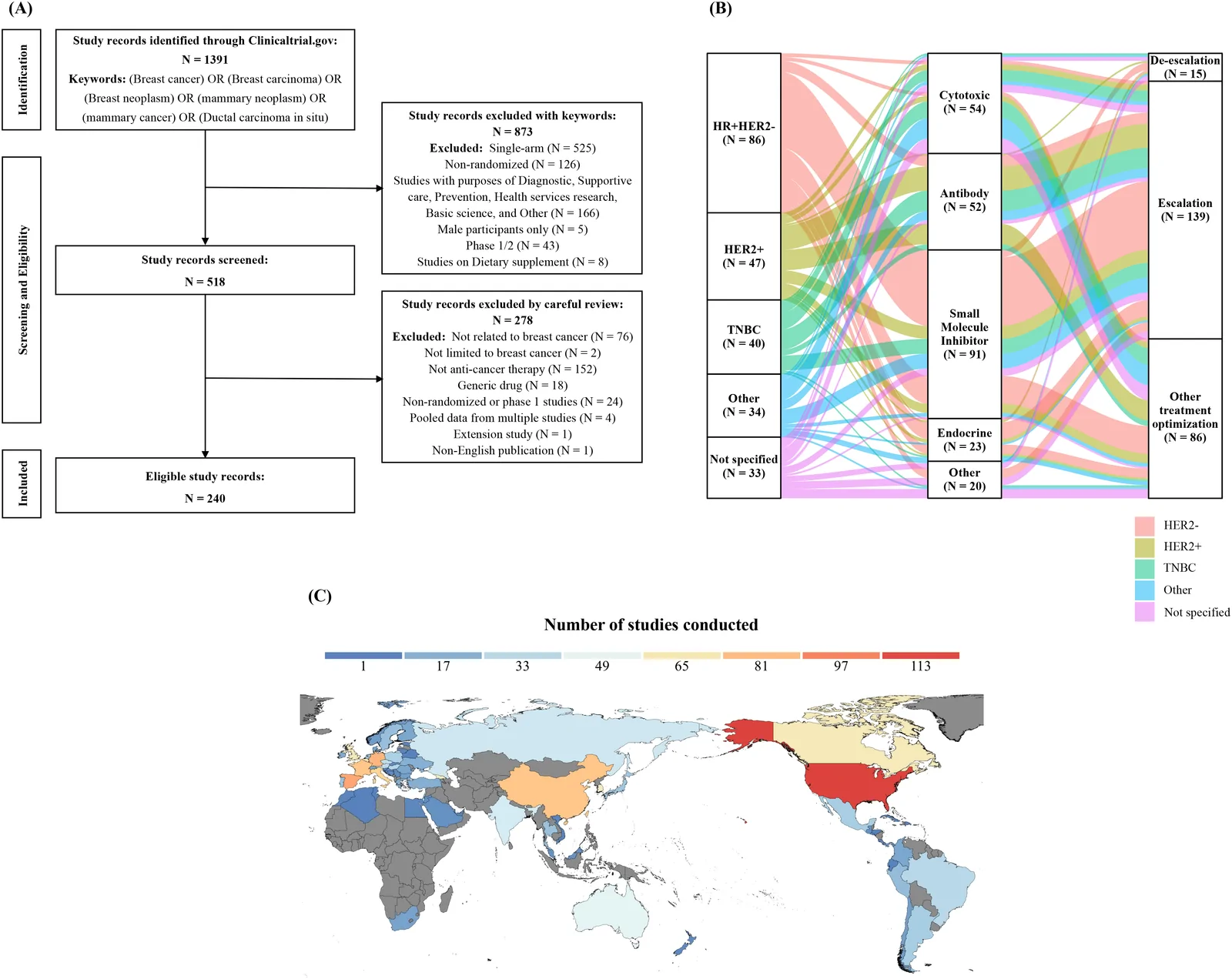
Overview of the study selection and characteristics of the included studies. **(A)** Study flow diagram; **(B)** alluvial diagram illustrating the distribution of breast cancer subtypes, intervention types, and study types among the included studies; **(C)** number of studies by country.

**Table 1:** Study characteristics stratified by study types.

Characteristic	Overall (N = 240)	De-escalation studies (N = 15)	Escalation studies (N = 139)	P value	Other treatment optimization studies (N = 86)	P value
Year of study start				0.551		0.769
2011-2015	158 (65.8)	9 (60.0)	94 (67.6)		55 (64.0)	
2016-2021	82 (34.2)	6 (40.0)	45 (32.4)		31 (36.0)	
Intervention				0.001		0.039
Pharmacological	228 (95.0)	11 (73.3)	137 (98.6)		80 (93.0)	
Nonpharmacological	12 (5.0)	4 (26.7)	2 (1.4)		6 (7.0)	
Phase				0.411		0.161
Phase 2	142 (59.2)	11 (73.3)	87 (62.6)		44 (51.2)	
Phase 2/3, 3 or 4	98 (40.8)	4 (26.7)	52 (37.4)		42 (48.8)	
Intent				0.014		0.378
Curative	90 (37.5)	9 (60.0)	40 (28.8)		41 (47.7)	
Palliative	150 (62.5)	6 (40.0)	99 (71.2)		45 (52.3)	
Blinding				0.010		0.386
Open-label	178 (74.2)	14 (93.3)	80 (57.6)		84 (97.7)	
Double-blinded	62 (25.8)	1 (6.7)	59 (42.4)		2 (2.3)	
Recruitment				0.001		0.047
National	120 (50.0)	13 (86.7)	56 (40.3)		51 (59.3)	
International	120 (50.0)	2 (13.3)	83 (59.7)		35 (40.7)	
Estimated enrollment				0.405		0.312
Small (≤100)	54 (22.5)	4 (26.7)	30 (21.6)		20 (23.3)	
Medium (101 to 300)	108 (45.0)	8 (53.3)	66 (47.5)		34 (39.5)	
Large (>300)	78 (32.5)	3 (20.0)	43 (30.9)		32 (37.2)	
Funding				<0.001		0.023
Non-industry	73 (30.4)	11 (73.3)	28 (20.1)		34 (39.5)	
Industry	167 (69.6)	4 (26.7)	111 (79.9)		52 (60.5)	

Among the 207 studies that specified the tumor subtype, the majority (41.5%, N = 86) investigated HR-positive/HER2-negative breast cancer, and the most frequently studied intervention for this subtype was small-molecule inhibitors (65.1%, 56 of 86). Antibodies were predominant in HER2-positive breast cancer studies (53.2%, 25 of 47), whereas cytotoxic chemotherapy was most common in TNBC studies (40%, 16 of 40) (**Figure 1B**). Overall, most participants were recruited from the United States (47.9%, 115 of 240), followed by Spain (36.7%, 88 of 240), and Germany (34.6%, 83 of 240) (**Figure 1C**).

Based on the experimental and control group interventions, only 15 of the 240 studies (6.3%) were classified as de-escalation studies, 139 (57.9%) as escalation studies, and 86 (35.8%) as other treatment optimization studies. Among the 15 de-escalation studies, 6 involved dose reduction, 4 involved shortened duration of treatment, and 5 involved chemotherapy omission or de-intensification. Of the 139 escalation studies, only 2 involved dose escalation, whereas the rest investigated treatment intensification. Among the 86 other treatment optimization studies, 72 compared two modalities head-to-head, 8 optimized the route of administration, 4 optimized the treatment sequence, and 2 optimized the treatment scheduling (**Supplementary Table 2**).

Regardless of the trial type, HR-positive/HER2-negative disease was the predominant subtype, accounting for 33.3% (5 of 15), 42.4% (59 of 139), and 25.6% (22 of 86) of de-escalation, escalation, and other treatment optimization studies, respectively. Small-molecule inhibitors were the most frequently investigated interventions in de-escalation and escalation trials (26.7% [4 of 15] and 46.0% [64 of 139], respectively), whereas cytotoxic chemotherapy was the most common intervention in other treatment optimization studies (38.4% [33 of 86]) (**Figure 1B**). Chemotherapy and radiotherapy were also commonly studied as interventions in the de-escalation studies (both 26.7% [4 of 15]).

### Study discontinuation

Forty-five of 240 studies (18.8%) were discontinued prematurely, with a median difference of 90 between the target and actual sample sizes. Among these, poor recruitment was the most common reason (40.0%, N = 18), followed by resources (17.8%, N = 8) and futility (15.6%, N = 7) (**Supplementary Table 3**). Premature discontinuation was more common in studies with palliative intent than in those with curative intent (23.3% vs. 11.1%, P = 0.019) (**Figure 2**).

**Figure 2:**
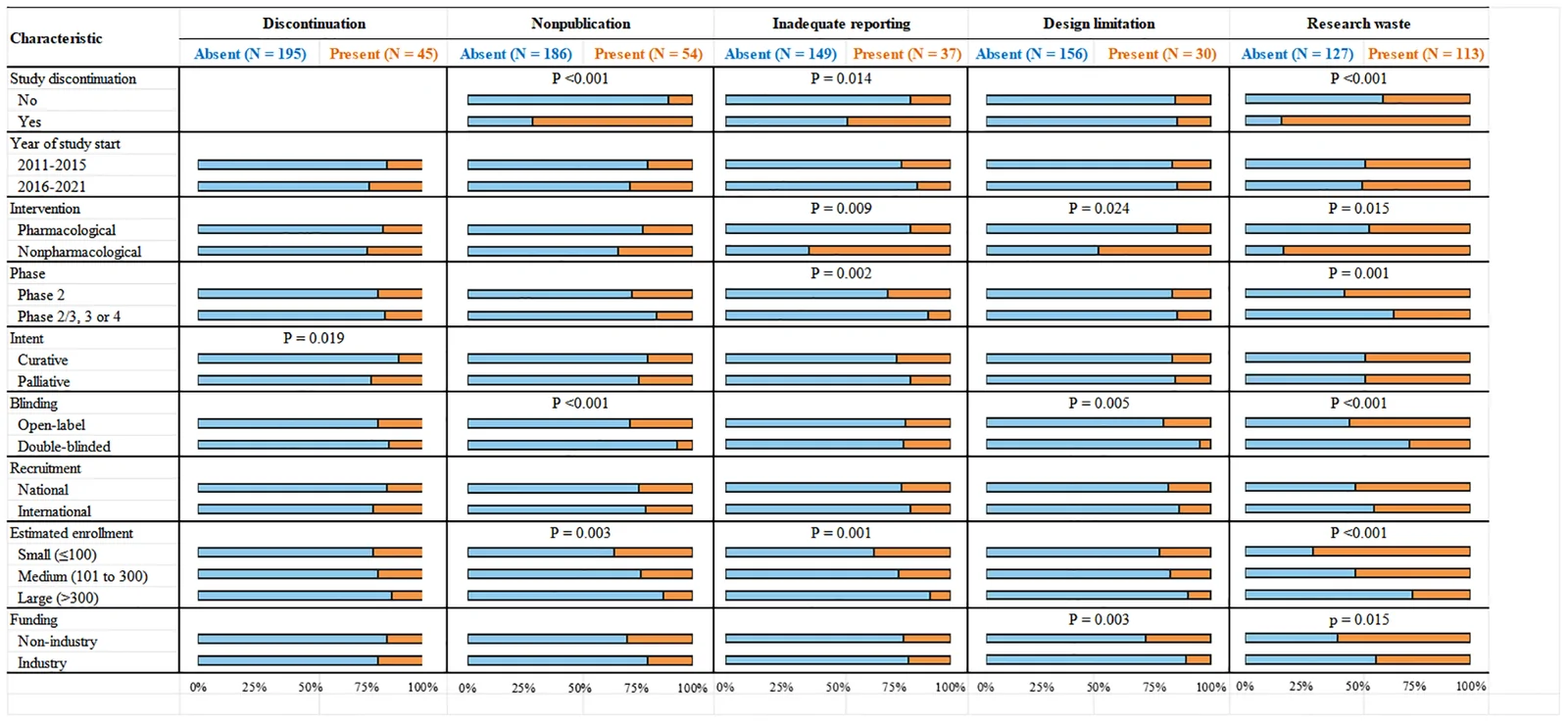
Associations between study characteristics and study discontinuation, non-publication, inadequate reporting, design limitation, and research waste.

### Research waste

Overall, 186 of the 240 studies (77.5%) were published in peer-reviewed journals, and the median time from study completion to publication was 21.5 months. **Figure 3A** depicts the nine journals with the highest publication frequencies, led by *The Lancet Oncology* (n = 28) and the *Journal of Clinical Oncology* (n = 23). Unpublished studies were more likely to be prematurely discontinued (59.3% vs. 7.0%, P <0.001), had an open-label design (92.6% vs, 68.8% P <0.001), and had a smaller sample size (P = 0.003) than published studies (**Figure 2**).

**Figure 3:**
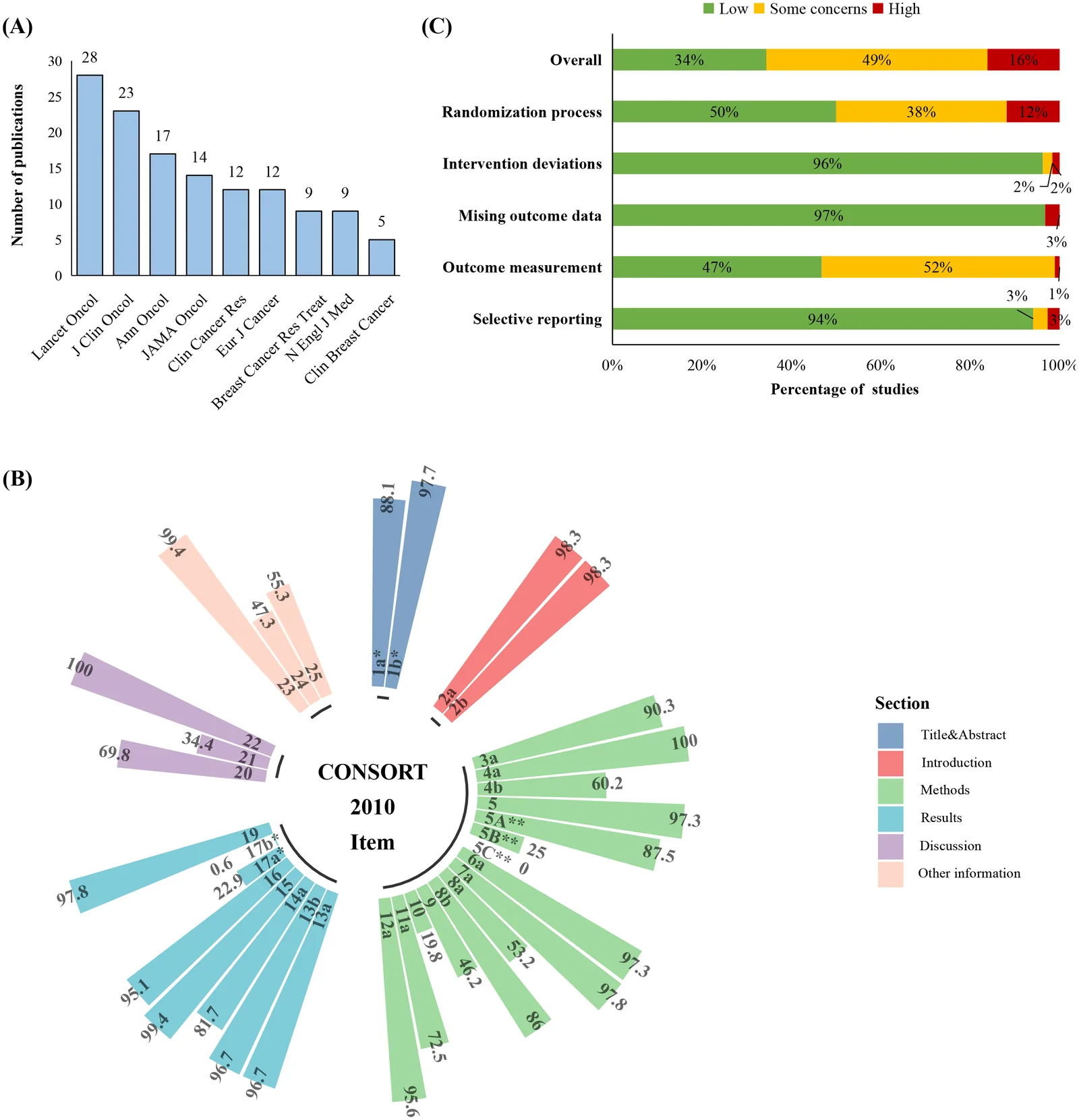
Overview of three components of research waste. **(A)** Top nine journals with the most publications; **(B)** compliance with items of the CONSORT 2010 checklist; **(C)** assessment of risk of bias.

Among 186 published studies, 149 (80.1%) were adequately reported. Inadequate reporting was primarily observed in outcome presentation (22.9% and 0.6% for items 17a and 17b, respectively) and implementation of non-pharmacological interventions (25% and 0% for items 5B and 5C, respectively) (**Figure 3B**). Inadequate reporting was more frequently observed in premature discontinuation (16.2% vs. 4.7%, P = 0.014), non-pharmacological (13.5% vs. 2.0%, P = 0.009), phase 2 (78.4% vs. 50.3%, P = 0.002), and small-scale (P = 0.001) studies (**Figure 2**). Moreover, 30 of the 186 published studies (16.1%) had design limitations, most frequently arising from the randomization process (11.8%, N = 22) (**Figure 3C**). Studies that examined nonpharmacological interventions (13.3% vs. 2.6%, P = 0.024), had an open-label design (90.0% vs. 64.7%, P = 0.005), or did not receive funding from the industry (50.0% vs. 23.7%, P = 0.003) were more likely to exhibit design limitations (**Figure 2**).

Overall, 113 of the 240 studies (47.1%) included at least one feature of research waste. Apart from the 54 unpublished studies, 59 of the published studies demonstrated research waste due to inadequate reporting or design limitations, including 8 studies with both deficiencies. Factors significantly associated with research waste included premature discontinuation (33.6% vs. 5.5%, P <0.001), non-pharmacological interventions (8.8% vs. 1.6%, P = 0.015), open-label (85.0% vs. 64.6%, P <0.001), phase 2 (70.8% vs. 48.8%, P <0.001), non-industry funding (38.1% vs. 23.6%, P = 0.015), and smaller sample size (P <0.001) (**Figure 2**). Multivariate analysis revealed that premature discontinuation (OR, 9.227; 95% CI, 3.639–23.398; P <0.001) was an independent risk factor for research waste, whereas a double-blind design (OR, 0.399; 95% CI, 0.182–0.872; P = 0.021) and large sample size (OR: 0.192, 95% CI: 0.062–0.596, P = 0.016) were independent protectors for research waste (**Supplementary Table 4**).

### Comparison of different study types

Compared to escalation and other treatment optimization studies, de-escalation studies were more likely to investigate non-pharmacological interventions (26.7% vs. 1.4%, P = 0.001; vs. 7.0%, P = 0.039), recruit participants from a single nation (86.7% vs. 40.3%, P = 0.001; vs. 59.3%, P = 0.047), and were less likely to be industry-funded (73.3% vs. 20.1%, P <0.001; vs. 39.5%, P = 0.023). Escalation studies more frequently examined palliative treatment (71.2% vs. 40.0%, P = 0.014) and were conducted with a double-blind design (42.4% vs. 6.7%, P = 0.010) than de-escalation studies (**Table 1**).

Escalation studies showed nearly two-fold higher rates of premature discontinuation (21.6% vs. 13.3%) and non-publication (24.5% vs. 13.3%) than de-escalation studies, alongside numerically lower rates of inadequate reporting (24.8% vs. 30.8%) and design deficiencies (13.3% vs. 15.4%) (**Figure 4A**). **Supplementary Table 3** shows the reasons for premature discontinuation across different study types. Notably, 2 discontinued de-escalation studies were attributed to poor recruitment. After adjusting for other factors, an escalation design was independently associated with an increased risk of research waste compared to a de-escalation design (OR, 4.337; 95% CI, 1.165–18.567; P = 0.028) (**Figure 4B**).

**Figure 4:**
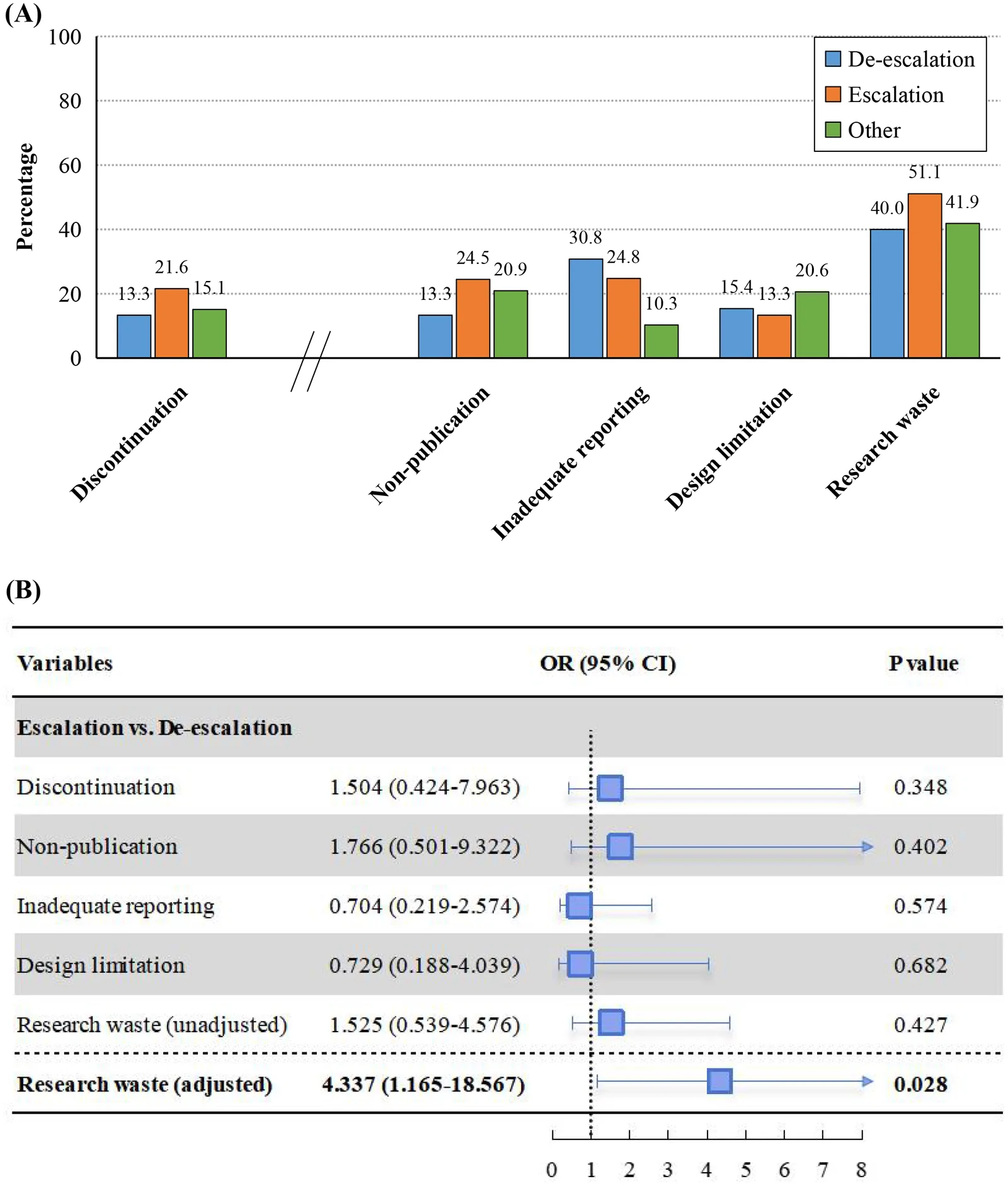
**(A)** Study discontinuation, non-publication, inadequate reporting, design limitation, and research waste stratified by study types. **(B)** Odd ratios (ORs) and 95% confidence intervals (CIs) comparing escalation versus de-escalation studies. Adjusted model includes study type, study discontinuation, intervention, phase, blinding, recruitment, estimated enrollment, and funding as covariates.

## Discussion

Our analysis indicated a substantial burden of research waste among phase 2 or higher RCTs evaluating systemic or local treatments for breast cancer over the past decade. This is not the first study to report this phenomenon. An analysis of breast cancer clinical trials registered at ClinicalTrials.gov revealed that more than half of them remained unpublished and only a small proportion were published within 24 months of study completion (15). Our study included only interventional RCTs with a nonpublication rate of less than one quarter. Based on a previously proposed definition (12), we assessed the incidence of inadequate reporting and design limitations. Considering these three factors together, nearly half of the studies exhibited research waste. Interestingly, escalation studies appear to be more prone to research waste than de-escalation studies. However, due to the small de-escalation cohort (N = 15) and the uncertainty in adjusted estimates, this result should be interpreted with caution.

Recently, marked improvements have been achieved in the cure rates of breast cancer, shifting attention toward the development of personalized medicine with the goal of tailoring treatment strategies without compromising efficacy (16). The increasingly refined molecular subtypes of breast cancer allow for stratified modulation of treatment planning. Modulations include escalation therapy for patients with adverse biological features or very high risk and de-escalation therapy for patients with favorable biology or lower risk. Specifically, de-escalation aims to achieve comparable survival benefits while reducing patient burden, minimizing adverse effects, and improving the long-term quality of life (9). As our results indicate, such studies have rarely been initiated by pharmaceutical companies as their results may undermine the commercial potential of the products. In contrast, escalation studies and other treatment optimization studies were predominantly industry-sponsored and more likely to be pharmacological and international.

Contrary to our initial hypothesis, de-escalation studies were independently associated with a reduced risk of research waste compared to escalation studies. When considering the components of research waste, de-escalation studies demonstrated an advantage in terms of publication rates; however, they performed less favorably than escalation studies in terms of reporting quality and study design. One possible explanation is the generally favorable attitude toward de-escalation therapies across the scientific community and society, encompassing care providers, patients, and their families, as well as government authorities, policymakers, and journal editors (17, 18). However, de-escalation studies may face funding constraints, which can limit access to specialized statistical consultation and professional medical writing support.

Identifying the risk factors of research waste is crucial for developing strategies to mitigate it. First, consistent with previous studies, premature discontinuation may be the most important factor in research waste, particularly because of its strong association with increased non-publication rates (19, 20). Regardless of study type, poor recruitment was the leading cause of study discontinuation, particularly preventable discontinuation. This is often due to investigators overestimating the eligible participants, highlighting the importance of accurately assessing the potential of each site prior to trial initiation (21). Continuous monitoring during the study, along with measures such as adding sites or reallocating resources, can help ensure that the enrollment targets are met (22). Limited resources constituted another major reason for the discontinuation of escalation studies. For economic reasons, pharmaceutical companies may prematurely withdraw funding from studies that are unlikely to generate substantial financial returns (23). Implementing transparency mandates and review mechanisms may help to mitigate premature discontinuation driven by commercial considerations (24). Moreover, large-scale studies are associated with reduced research waste, as they carry higher expectations from both investigators and sponsors, and their results are more likely to be published by journal editors (25). However, indiscriminate increases in the sample size are not justified and are inconsistent with our viewpoint. Although a double-blind design was identified as an important protective factor against research waste, it was not feasible in all the settings. Double-blinding is closely linked to a rigorous study design and adequate resources. For non-industry-funded studies, collaboration with academic clinical trial units may provide the necessary logistical and methodological support (26).

Under a broad definition, both de-escalation and escalation studies can be classified as treatment optimization studies (10). In this study, de-escalation, escalation, and other types of treatment optimization studies were examined separately. Other treatment optimization strategies typically involve substituting a less toxic and more effective agent (e.g., antibodies) for the standard agent (e.g., chemotherapy) or modifying conventional treatment delivery, such as the route, timing, or sequencing, to improve outcomes. These studies share characteristics that are similar to those of escalation studies; however, their burden of research waste more closely resembles that observed in de-escalation studies. Notably, these studies exhibited a higher proportion of design limitations than both the de-escalation and escalation studies, and the underlying mechanisms warrant further investigation.

Moreover, this study identified potential concerns regarding global inequity (27). As shown in **Figure 1B**, few RCTs have been conducted in Africa and Central Asia, limiting the generalizability of the study findings to diverse populations. Addressing these disparities will require coordinated multi-stakeholder collaboration and strategic resource allocation to strengthen workforce capacity, regulatory frameworks, ethical review processes, and data infrastructure (2).

This study has several strengths. As research waste can occur at different stages of the research cycle (28–30), we employed an all-or-none metric to assess it. Additionally, we modified the definition proposed by Chapman et al. to enhance its robustness. Furthermore, we examined, for the first time, the impact of the study type on research waste, thereby providing valuable evidence for future improvement strategies.

This study has several limitations. First, a relatively small proportion of de-escalation studies was identified, which reduced the statistical power. Second, research waste is a multidimensional concept that extends beyond the definitions adopted in this study. For example, in de-escalation studies, the absence of a properly predefined non-inferiority margin can render the results difficult to incorporate into guidelines (16), which also constitutes waste. Third, all the included studies were drawn from a single registry (ClinicalTrials.gov), which may limit the generalizability of our findings. However, other registries also have significant weaknesses such as incomplete data and publications in non-English journals, making data aggregation challenging. Fourth, this study included only “carefully selected” clinical trials, representing a small fraction of all breast cancer research. Therefore, the actual burden of research waste may be greater. Fifth, the exclusion of several representative de-escalation studies (e.g., the SENOMAC study (31)) due to specific eligibility criteria may have impacted the results. Finally, our findings should be interpreted as exploratory and hypothesis-generating rather than confirmatory.

## Conclusions

This study revealed the current status of research waste in phase 2 to 4 RCTs on breast cancer treatment and reported differences in research waste among de-escalation, escalation, and other treatment optimization studies. Future efforts should focus on reducing preventable premature discontinuation and on enhancing study recruitment and design. Despite substantial efforts, the current evaluations of research waste remain limited in scope and depth, and are subject to inherent statistical instability, highlighting the need for further comprehensive investigations.

## Data Availability

The raw data supporting the conclusions of this article will be made available by the authors, without undue reservation.

## References

[1] Gargon E Gorst SL Harman NL Smith V Matvienko-Sikar K Williamson PR . Choosing important health outcomes for comparative effectiveness research: 4th annual update to a systematic review of core outcome sets for research. PloS One. (2018) 13:e0209869. doi: 10.1371/journal.pone.0209869 30592741 PMC6310275

[2] Casolino R Tatah L Charnaud S Santero M Ilbawi A Ross AL . The WHO global landscape of cancer clinical trials. Nat Med. (2025) 31:2901–12. doi: 10.1038/s41591-025-03926-x 40926101

[3] Al-Shahi Salman R Beller E Kagan J Hemminki E Phillips RS Savulescu J . Increasing value and reducing waste in biomedical research regulation and management. Lancet (London England). (2014) 383:176–85. doi: 10.1016/s0140-6736(13)62297-7 24411646 PMC3952153

[4] Chalmers I Glasziou P . Avoidable waste in the production and reporting of research evidence. Lancet (London England). (2009) 374:86–9. doi: 10.1097/aog.0b013e3181c3020d 19525005

[5] Ioannidis JP . How to make more published research true. PloS Med. (2014) 11:e1001747. doi: 10.1371/journal.pmed.1001747 25334033 PMC4204808

[6] Lu J Xu B Shen L Wu D Xue Z Zheng H . Characteristics and research waste among randomized clinical trials in gastric cancer. JAMA Netw Open. (2021) 4:e2124760. doi: 10.1001/jamanetworkopen.2021.24760 34533573 PMC8449283

[7] Lin L Tang Y Yang L Wang Y Chen R . Research waste among randomized controlled trials in ovarian cancer: A cross-sectional study. Eur J Surg Oncol. (2024) 50:108437. doi: 10.1016/j.ejso.2024.108437 38820926

[8] Chen H Sun B Hua C Lin X . Characteristics and research waste of randomized controlled trials in melanoma. Clin Exp Dermatol. (2024) 49:1611–8. doi: 10.1093/ced/llae248 38913453

[9] Trapani D Franzoi MA Burstein HJ Carey LA Delaloge S Harbeck N . Risk-adapted modulation through de-intensification of cancer treatments: an ESMO classification. Ann Oncol. (2022) 33:702–12. doi: 10.1016/j.annonc.2022.03.273 35550723

[10] Saesen R Lacombe D Huys I . Design, organisation and impact of treatment optimisation studies in breast, lung and colorectal cancer: The experience of the European Organisation for Research and Treatment of Cancer. Eur J Cancer. (2021) 151:221–32. doi: 10.1016/j.ejca.2021.04.012 34023561

[11] Cao L Sugumar K Keller E Li P Rock L Simpson A . Neoadjuvant endocrine therapy as an alternative to neoadjuvant chemotherapy among hormone receptor-positive breast cancer patients: Pathologic and surgical outcomes. Ann Surg Oncol. (2021) 28:5730–41. doi: 10.1245/s10434-021-10459-3 34342757 PMC8330206

[12] Chapman S Aldaffaa M Downey C Jayne D . Research waste in surgical randomized controlled trials. Br J Surg. (2019) 106:1464–71. doi: 10.1002/bjs.11266 31393612

[13] Sterne JAC Savović J Page MJ Elbers RG Blencowe NS Boutron I . RoB 2: a revised tool for assessing risk of bias in randomised trials. Bmj. (2019) 366:l4898. doi: 10.1136/bmj.l4898 31462531

[14] Speich B Gryaznov D Busse JW Gloy VL Lohner S Klatte K . Nonregistration, discontinuation, and nonpublication of randomized trials: A repeated metaresearch analysis. PloS Med. (2022) 19:e1003980. doi: 10.1371/journal.pmed.1003980 35476675 PMC9094518

[15] Asiimwe IG Rumona D . Publication proportions for registered breast cancer trials: before and following the introduction of the ClinicalTrials.gov results database. Res Integrity Peer Rev. (2016) 1:10. doi: 10.1186/s41073-016-0017-4 29451530 PMC5803577

[16] Piccart MJ Hilbers FS Bliss JM Caballero C Frank ES Renault P . Road map to safe and well-designed de-escalation trials of systemic adjuvant therapy for solid tumors. J Clin Oncol. (2020) 38:4120–9. doi: 10.1200/jco.20.01382 33052755

[17] Ferrero JM Bouriga R Gal J Milano G . De-escalating anticancer treatment: Watch your step. Cancers (Basel). (2025) 17(15):2474. doi: 10.3390/cancers17152474 40805173 PMC12346573

[18] Hamilton R Hashemi E Uthayakumar A Liang M Mayo S Parker K . Patient and family caregiver perspectives on therapy de-escalation in cancer: A scoping review. Psycho-oncology. (2025) 34:e70104. doi: 10.1002/pon.70104 39971709 PMC11839414

[19] Lu D Chen X Mu Y Kong L Zhang L Li J . Discontinuation and non-publication of randomized controlled trials on cervical cancer or precancer. Jpn J Clin Oncol. (2024) 54:1141–9. doi: 10.1093/jjco/hyae096 39041316

[20] Chen H Lin K Dai Z Pan S . Clinical trials in small-cell lung cancer treatment: a cross-sectional analysis of trial discontinuation and non-publication. Discover Oncol. (2025) 16:2169. doi: 10.1007/s12672-025-03967-1 41296130 PMC12657696

[21] McDonald AM Knight RC Campbell MK Entwistle VA Grant AM Cook JA . What influences recruitment to randomised controlled trials? A review of trials funded by two UK funding agencies. Trials. (2006) 7:9. doi: 10.1186/1745-6215-7-9 16603070 PMC1475627

[22] Kaur G Smyth RL Williamson P . Developing a survey of barriers and facilitators to recruitment in randomized controlled trials. Trials. (2012) 13:218. doi: 10.1186/1745-6215-13-218 23171513 PMC3563446

[23] Psaty BM Rennie D . Stopping medical research to save money: a broken pact with researchers and patients. Jama. (2003) 289:2128–31. doi: 10.1001/jama.289.16.2128 12709471

[24] Bruckner T Rodgers F Styrmisdóttir L Keestra S . Adoption of World Health Organization best practices in clinical trial transparency among European medical research funder policies. JAMA Netw Open. (2022) 5:e2222378. doi: 10.1001/jamanetworkopen.2022.22378 35913742 PMC9344358

[25] Chapman SJ Shelton B Mahmood H Fitzgerald JE Harrison EM Bhangu A . Discontinuation and non-publication of surgical randomised controlled trials: observational study. Bmj. (2014) 349:g6870. doi: 10.1136/bmj.g6870 25491195 PMC4260649

[26] Wang F Wang X Liu E Song F Chen Y . Impacts of clinical research units on clinical research - a systematic review of empirical studies. Systematic Rev. (2025) 14:94. doi: 10.1016/b978-0-12-818381-6.00002-4 40296152 PMC12039282

[27] Wells JC Sharma S Del Paggio JC Hopman WM Gyawali B Mukherji D . An analysis of contemporary oncology randomized clinical trials from low/middle-income vs high-income countries. JAMA Oncol. (2021) 7:379–85. doi: 10.1001/jamaoncol.2020.7478 33507236 PMC7844695

[28] Chan A Song F Vickers A Jefferson T Dickersin K Gøtzsche PC . Increasing value and reducing waste: addressing inaccessible research. Lancet (London England). (2014) 383:257–66. doi: 10.1016/s0140-6736(13)62296-5 24411650 PMC4533904

[29] Glasziou P Altman DG Bossuyt P Boutron I Clarke M Julious S . Reducing waste from incomplete or unusable reports of biomedical research. Lancet. (2014) 383:267–76. doi: 10.1016/s0140-6736(13)62228-x 24411647

[30] Ioannidis J Greenland S Hlatky M Khoury M Macleod M Moher D . Increasing value and reducing waste in research design, conduct, and analysis. Lancet (London England). (2014) 383:166–75. doi: 10.1016/s0140-6736(13)62227-8 24411645 PMC4697939

[31] de Boniface J Filtenborg Tvedskov T Rydén L Szulkin R Reimer T Kühn T . Omitting axillary dissection in breast cancer with sentinel-node metastases. N Engl J Med. (2024) 390:1163–75. doi: 10.1056/nejmoa2313487 38598571

