# Supplementary material for: Profiles of research waste in randomized clinical trials comparing de-escalation and escalation strategies for breast cancer treatment
Source: Front Oncol. 2026 Sep 24;16:1935566. doi: 10.3389/fonc.2026.1935566 (PMC13645710; doi:10.3389/fonc.2026.1935566)
Supplement: Supplementary file 1 [file Table1.doc]

Table S1. Consolidated Standards of Reporting Trials (CONSORT) 2010 checklist

| **No** | **Item** |
| --- | --- |
| 1a | Identification as a randomised trial in the title |
| 1b | Structured summary of trial design, methods, results, and conclusions |
| 2a | Scientific background and explanation of rationale |
| 2b | Specific objectives or hypotheses |
| 3a | Description of trial design (such as parallel, factorial) including allocation ratio |
| 3b* | Important changes to methods after trial commencement (such as eligibility criteria), with reasons |
| 4a | Eligibility criteria for participants |
| 4b | Settings and locations where the data were collected |
| 5 | The interventions for each group with sufficient details to allow replication |
| 5A** | Description of the components of the interventions and, if applicable, the procedure for individualizing treatment |
| 5B** | Details of how the interventions were standardized |
| 5C** | Details of how the adherence of care provers with the protocol was assessed or enhanced |
| 6a | Completely defined pre-specified primary and secondary outcome measures |
| 6b* | Any changes to trial outcomes after the trial commenced, with reasons |
| 7a | How sample size was determined |
| 7b* | When applicable, explanation of any interim analyses and stopping guidelines |
| 8a | Method used to generate the random allocation sequence |
| 8b | Type of randomisation; details of any restriction (such as blocking and block size) |
| 9 | Mechanism used to implement the random allocation sequence |
| 10 | Who generated the random allocation sequence, enrolled participants, and assigned participants to interventions |
| 11a | If done, who was blinded after assignment to interventions and how |
| 11b* | If relevant, description of the similarity of interventions |
| 12a | Statistical methods used to compare groups for primary and secondary outcomes |
| 12b* | Methods for additional analyses, such as subgroup analyses and adjusted analyses |
| 13a | The numbers of participants who were randomised, received treatment, and analysed for the primary outcome |
| 13b | For each group, losses and exclusions after randomisation, together with reasons |
| 14a | Dates defining the periods of recruitment and follow-up |
| 14b* | Why the trial ended or was stopped |
| 15 | A table showing baseline demographic and clinical characteristics for each group |
| 16 | For each group, number of participants analysed and whether the analysis was by original assigned groups |
| 17a | For each primary and secondary outcome, results for each group, and the estimated effect size and its precision |
| 17b | For binary outcomes, presentation of both absolute and relative effect sizes is recommended |
| 18* | Results of any other analyses performed, including subgroup analyses and adjusted analyses |
| 19 | All important harms or unintended effects in each group |
| 20 | Trial limitations, addressing sources of potential bias, imprecision, and, if relevant, multiplicity of analyses |
| 21 | Generalisability (external validity, applicability) of the trial findings |
| 22 | Interpretation consistent with results, balancing benefits and harms, and considering other relevant evidence |
| 23 | Registration number and name of trial registry |
| 24 | Where the full trial protocol can be accessed, if available |
| 25 | Sources of funding and other support (such as supply of drugs), role of funders |
| * indicates a conditional item for which not all manuscripts were scored. ** items relate to non-pharmacological studies only. | |

**Table S2.** Characteristics and classification rationale of included studies

| **No** | **Registry ID** | **Brief Title** | Study focus ● De-esca1ation **● Esca1ation ● Other treatment optimization** | **Research waste  and its components** |
| --- | --- | --- | --- | --- |
| 1 | NCT01160211 | A Study to Compare the Safety and Efficacy of an Aromatase Inhibitor in Combination With Lapatinib, Trastuzumab or Both for the Treatment of Hormone Receptor Positive, HER2+ Metastatic Breast Cancer | **Add therapeutic component** | × Research waste × Nonpublication × Inadequate reporting × Design limitations |
| 2 | NCT01186991 | Study Evaluating the Safety and Efficacy of Onartuzumab And/or Bevacizumab in Combination With Paclitaxel in Participants With Metastatic, Triple Negative Breast Cancer | **Add therapeutic component** | × Research waste × Nonpublication × Inadequate reporting × Design limitations |
| 3 | NCT01216111 | Adjuvant Platinum and Taxane in Triple-negative Breast Cancer (PATTERN) | **Compare different modalities** | × Research waste × Nonpublication × Inadequate reporting × Design limitations |
| 4 | NCT01233947 | Study of AFP464 +/- Faslodex in ER + Breast Cancer | **Add therapeutic component** | √ Research waste √ Nonpublication Inadequate reporting Design limitations |
| 5 | NCT01234337 | Phase III Trial Comparing Capecitabine in Combination With Sorafenib or Placebo in the Treatment of Locally Advanced or Metastatic HER2-Negative Breast Cancer | **Add therapeutic component** | √ Research waste × Nonpublication √ Inadequate reporting × Design limitations |
| 6 | NCT01234402 | Study of Icrucumab (IMC-18F1) or Ramucirumab Drug Product (DP) in Combination With Capecitabine or Capecitabine on Previously Treated Breast Cancer Patients | **Add therapeutic component** | √ Research waste × Nonpublication √ Inadequate reporting × Design limitations |
| 7 | NCT01250379 | A Study of Avastin (Bevacizumab) in Combination With Chemotherapy in Patients With Breast Cancer Progressing After First-Line Therapy With Avastin and Chemotherapy (TANIA) | **Add therapeutic component** | × Research waste × Nonpublication × Inadequate reporting × Design limitations |
| 8 | NCT01275677 | Chemotherapy With or Without Trastuzumab After Surgery in Treating Women With Invasive Breast Cancer | **Add therapeutic component** | × Research waste × Nonpublication × Inadequate reporting × Design limitations |
| 9 | NCT01287624 | Gemcitabine Plus Cisplatin Versus Gemcitabine Plus Paclitaxel in Triple Negative Breast Cancer (TNBC) | **Compare different modalities** | × Research waste × Nonpublication × Inadequate reporting × Design limitations |
| 10 | NCT01300351 | Comparing the Efficacy and Tolerability of Fulvestrant 500 mg Versus 250 mg in Advanced Breast Cancer Women | **Increase cumulative dose** | × Research waste × Nonpublication × Inadequate reporting × Design limitations |
| 11 | NCT01307891 | Abraxane With or Without Tigatuzumab in Patients With Metastatic, Triple Negative Breast Cancer | **Add therapeutic component** | √ Research waste × Nonpublication √ Inadequate reporting × Design limitations |
| 12 | NCT01358877 | A Study of Pertuzumab in Addition to Chemotherapy and Trastuzumab as Adjuvant Therapy in Participants With Human Epidermal Growth Receptor 2 (HER2)-Positive Primary Breast Cancer | **Add therapeutic component** | × Research waste × Nonpublication × Inadequate reporting × Design limitations |
| 13 | NCT01367028 | A Phase II Study of Neoadjuvant Trastuzumab+Docetaxel+NPLD+/-Bevacizumab in Her2-pos. Early Breast Cancer | **Add therapeutic component** | √ Research waste √ Nonpublication Inadequate reporting Design limitations |
| 14 | NCT01381874 | A Study of Abiraterone Acetate Plus Prednisone With or Without Exemestane in Postmenopausal Women With Estrogen Receptor-Positive (ER+) Metastatic Breast Cancer Progressing After Letrozole or Anastrozole Therapy | **Add therapeutic component** | √ Research waste × Nonpublication √ Inadequate reporting × Design limitations |
| 15 | NCT01392586 | Systemic Therapy With or Without Upfront Surgery in Metastatic Breast Cancer | **Add therapeutic component** | √ Research waste √ Nonpublication Inadequate reporting Design limitations |
| 16 | NCT01413828 | Trastuzumab Administered Concurrently or Sequentially to Anthracycline-containing Adjuvant Regimen for Breast Cancer | **Compare different sequences** | × Research waste × Nonpublication × Inadequate reporting × Design limitations |
| 17 | NCT01416389 | A Study of LY2523355 in Participants With Breast Cancer | **Add therapeutic component** | √ Research waste √ Nonpublication Inadequate reporting Design limitations |
| 18 | NCT01419197 | A Study of Trastuzumab Emtansine in Comparison With Treatment of Physician's Choice in Participants With HER2-positive Breast Cancer Who Have Received at Least Two Prior Regimens of HER2-directed Therapy | **Compare different modalities** | × Research waste × Nonpublication × Inadequate reporting × Design limitations |
| 19 | NCT01421472 | A Trial of Preoperative MM-121 With Paclitaxel in HER2-negative Breast Cancer | **Add therapeutic component** | √ Research waste √ Nonpublication Inadequate reporting Design limitations |
| 20 | NCT01426880 | Addition of Carboplatin to Neoadjuvant Therapy for Triple-negative and HER2-positive Early Breast Cancer | **Add therapeutic component** | × Research waste × Nonpublication × Inadequate reporting × Design limitations |
| 21 | NCT01427933 | A Study of Ramucirumab (IMC-1121B) in Combination With Eribulin Versus Eribulin Alone in Participants With Breast Cancer | **Add therapeutic component** | × Research waste × Nonpublication × Inadequate reporting × Design limitations |
| 22 | NCT01430585 | Pre-Operative Study of PF-4691502 With Letrozole Compared To Letrozole Alone In Patients With Early Breast Cancer | **Add therapeutic component** | √ Research waste √ Nonpublication Inadequate reporting Design limitations |
| 23 | NCT01437566 | Study of GDC-0941 or GDC-0980 With Fulvestrant Versus Fulvestrant in Advanced or Metastatic Breast Cancer in Participants Resistant to Aromatase Inhibitor Therapy | **Add therapeutic component** | × Research waste × Nonpublication × Inadequate reporting × Design limitations |
| 24 | NCT01441596 | Lux-Breast 3; Afatinib Alone or in Combination With Vinorelbine in Patients With Human Epidermal Growth Factor Receptor 2 (HER2) Positive Breast Cancer Suffering From Brain Metastases | **Add therapeutic component** | × Research waste × Nonpublication × Inadequate reporting × Design limitations |
| 25 | NCT01491737 | A Study of Pertuzumab in Combination With Trastuzumab Plus an Aromatase Inhibitor in Participants With Metastatic Human Epidermal Growth Factor Receptor 2 (HER2)-Positive and Hormone Receptor-Positive Advanced Breast Cancer | **Add therapeutic component** | × Research waste × Nonpublication × Inadequate reporting × Design limitations |
| 26 | NCT01492101 | The BEACON Study (Breast Cancer Outcomes With NKTR-102) | **Compare different modalities** | × Research waste × Nonpublication × Inadequate reporting × Design limitations |
| 27 | NCT01506609 | Study Evaluating Efficacy And Tolerability Of Veliparib in Combination With Temozolomide (TMZ) or In Combination With Carboplatin and Paclitaxel Versus Placebo in Participants With Breast Cancer Gene (BRCA)1 and BRCA2 Mutation and Metastatic Breast Cancer | **Add therapeutic component** | × Research waste × Nonpublication × Inadequate reporting × Design limitations |
| 28 | NCT01516307 | Trial of Active Immunotherapy With Globo H-KLH (OPT-822) in Metastatic Breast Cancer Subjects | **Add therapeutic component** | × Research waste × Nonpublication × Inadequate reporting × Design limitations |
| 29 | NCT01520103 | Study to Compare Vinorelbine In Combination With the mTOR Inhibitor Everolimus vs. Vinorelbin Monotherapy for Second-line Treatment in Advanced Breast Cancer | **Add therapeutic component** | × Research waste × Nonpublication × Inadequate reporting × Design limitations |
| 30 | NCT01526369 | A Randomized Study of TH Versus THL in First Line Treatment of HER2-positive Metastatic Breast Cancer | **Add therapeutic component** | √ Research waste √ Nonpublication Inadequate reporting Design limitations |
| 31 | NCT01527487 | Trial of Eribulin/Cyclophosphamide or Docetaxel/Cyclophosphamide as Neoadjuvant Therapy in Locally Advanced HER2-Negative Breast Cancer | **Compare different modalities** | × Research waste × Nonpublication × Inadequate reporting × Design limitations |
| 32 | NCT01548677 | Efficacy Study of Herceptin to Treat HER2-negative CTC Breast Cancer | **Add therapeutic component** | × Research waste × Nonpublication × Inadequate reporting × Design limitations |
| 33 | NCT01572727 | A Study of the Experimental Drug BKM120 With Paclitaxel in Patients With HER2 Negative, Locally Advanced or Metastatic Breast Cancer, With or Without PI3K Activation | **Add therapeutic component** | √ Research waste × Nonpublication √ Inadequate reporting × Design limitations |
| 34 | NCT01583426 | Nanoparticle-based Paclitaxel vs Solvent-based Paclitaxel as Part of Neoadjuvant Chemotherapy for Early Breast Cancer (GeparSepto) | **Compare different modalities** | × Research waste × Nonpublication × Inadequate reporting × Design limitations |
| 35 | NCT01593020 | Neoadjuvant Study of Sequential Eribulin Followed by FAC Compared to Sequential Paclitaxel Followed by FEC in Early Stage Breast Cancer Not Overexpressing HER-2 | **Compare different modalities** | √ Research waste √ Nonpublication Inadequate reporting Design limitations |
| 36 | NCT01597414 | Elderly Metastatic Breast Cancer: Pertuzumab-Herceptin vs Pertuzumab-Herceptin-Metronomic Chemotherapy, Followed by T-DM1 | **Add therapeutic component** | √ Research waste × Nonpublication × Inadequate reporting √ Design limitations |
| 37 | NCT01605396 | A Phase II Trial of Ridaforolimus and Exemestane, Compared to Ridaforolimus, Dalotuzumab and Exemestane in Participants With Breast Cancer (MK-8669-064) | **Add therapeutic component** | × Research waste × Nonpublication × Inadequate reporting × Design limitations |
| 38 | NCT01610284 | Phase III Study of BKM120/Placebo With Fulvestrant in Postmenopausal Patients With Hormone Receptor Positive HER2-negative Locally Advanced or Metastatic Breast Cancer Refractory to Aromatase Inhibitor | **Add therapeutic component** | × Research waste × Nonpublication × Inadequate reporting × Design limitations |
| 39 | NCT01617668 | A Randomized, Phase 2, Neoadjuvant Study of Weekly Paclitaxel With or Without LCL161 in Patients With Triple Negative Breast Cancer | **Add therapeutic component** | √ Research waste × Nonpublication √ Inadequate reporting × Design limitations |
| 40 | NCT01619111 | DETECT III - A Multicenter, Phase III Study to Compare Standard Therapy +/- Lapatinib in HER2-ve MBC-Patients With HER2+ve CTCs | **Add therapeutic component** | √ Research waste × Nonpublication × Inadequate reporting √ Design limitations |
| 41 | NCT01622868 | Whole-Brain Radiation Therapy or Stereotactic Radiosurgery With or Without Lapatinib Ditosylate in Treating Patients With Brain Metastasis From HER2-Positive Breast Cancer | **Add therapeutic component** | √ Research waste × Nonpublication √ Inadequate reporting × Design limitations |
| 42 | NCT01633060 | A Phase III Study of BKM120 With Fulvestrant in Patients With HR+,HER2-, AI Treated, Locally Advanced or Metastatic Breast Cancer Who Progressed on or After mTORi | **Add therapeutic component** | × Research waste × Nonpublication × Inadequate reporting × Design limitations |
| 43 | NCT01644890 | A Phase III Study of NK105 in Patients With Breast Cancer | **Compare different modalities** | × Research waste × Nonpublication × Inadequate reporting × Design limitations |
| 44 | NCT01645839 | Interest of Intrathecal Chemotherapy With Liposomal Cytarabine (DepoCyte®) in Meningeal Metastasis of Breast Cancer | **Add therapeutic component** | √ Research waste × Nonpublication × Inadequate reporting √ Design limitations |
| 45 | NCT01656538 | A Study of Reolysin For Patients With Advanced/Metastatic Breast Cancer | **Add therapeutic component** | √ Research waste × Nonpublication × Inadequate reporting √ Design limitations |
| 46 | NCT01658462 | Phase II Study of Docetaxel +/- Nintedanib in Breast Cancer | **Add therapeutic component** | √ Research waste √ Nonpublication Inadequate reporting Design limitations |
| 47 | NCT01663727 | Study To Evaluate the Efficacy and Safety Of Bevacizumab, and Associated Biomarkers, In Combination With Paclitaxel Compared With Paclitaxel Plus Placebo as First-line Treatment Of Patients With Her2-Negative Metastatic Breast Cancer | **Add therapeutic component** | √ Research waste × Nonpublication √ Inadequate reporting × Design limitations |
| 48 | NCT01670500 | Cisplatin vs. Doxorubicin/Cyclophosphamide in BrCa | **Compare different modalities** | × Research waste × Nonpublication × Inadequate reporting × Design limitations |
| 49 | NCT01690702 | Study of Nab-Paclitaxel in High Risk Early Breast Cancer | **Compare different modalities** | × Research waste × Nonpublication × Inadequate reporting × Design limitations |
| 50 | NCT01698281 | Phase 2 Trial of AEZS-108 in Chemotherapy Refractory in Triple Negative Breast Cancer | **Compare different modalities** | √ Research waste √ Nonpublication Inadequate reporting Design limitations |
| 51 | NCT01705691 | Comparison of Neoadjuvant Chemotherapy With Weekly Paclitaxel or Eribulin Followed by A/C in Women With Locally Advanced HER2-Negative Breast Cancer | **Compare different modalities** | √ Research waste × Nonpublication √ Inadequate reporting × Design limitations |
| 52 | NCT01740336 | A Study of Paclitaxel With GDC-0941 Versus Paclitaxel With Placebo in Participants With Locally Recurrent or Metastatic Breast Cancer | **Add therapeutic component** | √ Research waste × Nonpublication √ Inadequate reporting × Design limitations |
| 53 | NCT01740427 | A Study of Palbociclib (PD-0332991) + Letrozole vs. Letrozole For 1st Line Treatment Of Postmenopausal Women With ER+/HER2- Advanced Breast Cancer (PALOMA-2) | **Add therapeutic component** | × Research waste × Nonpublication × Inadequate reporting × Design limitations |
| 54 | NCT01745367 | Tivozanib in Combination With Paclitaxel in Patients With Locally Recurrent or Metastatic Triple Negative Breast Cancer | **Add therapeutic component** | √ Research waste √ Nonpublication Inadequate reporting Design limitations |
| 55 | NCT01745965 | A Prospective, Randomized Multicenter, Open-label Comparison of Preoperative Trastuzumab Emtansine (T-DM1) With or Without Standard Endocrine Therapy vs. Trastuzumab With Standard Endocrine Therapy Given for Twelve Weeks in Patients With Operable HER2+/HR+ Breast Cancer Within the ADAPT Protocol. | **Compare different modalities** | √ Research waste × Nonpublication √ Inadequate reporting × Design limitations |
| 56 | NCT01772472 | A Study of Trastuzumab Emtansine Versus Trastuzumab as Adjuvant Therapy in Patients With HER2-Positive Breast Cancer Who Have Residual Tumor in the Breast or Axillary Lymph Nodes Following Preoperative Therapy (KATHERINE) | **Compare different modalities** | × Research waste × Nonpublication × Inadequate reporting × Design limitations |
| 57 | NCT01773460 | Everolimus Beyond Progress for Patients Who Had Progress Under Everolimus and Exemestane | **Add therapeutic component** | √ Research waste √ Nonpublication Inadequate reporting Design limitations |
| 58 | NCT01779050 | Effect of Trastuzumab on Disease Free Survival in Early Stage HER2-Negative Breast Cancer Patients With ERBB2 Expressing Disseminated Tumor Cells | **Add therapeutic component** | √ Research waste √ Nonpublication Inadequate reporting Design limitations |
| 59 | NCT01779479 | Efficacy and Safety of Cabazitaxel Versus Weekly Paclitaxel as Neo-adjuvant Treatment in Patients With Triple Negative or Luminal B/HER2 Normal BC (GENEVIEVE) | **Compare different modalities** | × Research waste × Nonpublication × Inadequate reporting × Design limitations |
| 60 | NCT01783444 | A Phase II Study of Everolimus in Combination With Exemestane Versus Everolimus Alone Versus Capecitabine in Advance Breast Cancer. | **Add therapeutic component** | √ Research waste × Nonpublication × Inadequate reporting √ Design limitations |
| 61 | NCT01792050 | Study of Chemotherapy in Combination With IDO Inhibitor in Metastatic Breast Cancer | **Add therapeutic component** | × Research waste × Nonpublication × Inadequate reporting × Design limitations |
| 62 | NCT01797120 | Study of Fulvestrant +/- Everolimus in Post-Menopausal, Hormone-Receptor + Metastatic Breast Ca Resistant to AI | **Add therapeutic component** | √ Research waste × Nonpublication √ Inadequate reporting × Design limitations |
| 63 | NCT01808573 | A Study of Neratinib Plus Capecitabine Versus Lapatinib Plus Capecitabine in Patients With HER2+ Metastatic Breast Cancer Who Have Received Two or More Prior HER2 Directed Regimens in the Metastatic Setting | **Compare different modalities** | × Research waste × Nonpublication × Inadequate reporting × Design limitations |
| 64 | NCT01810393 | A Study to Assess Preference for Subcutaneous Trastuzumab Treatment in Participants With Human Epidermal Growth Factor Receptor (HER)2-Positive Metastatic Breast Cancer Responding to First-Line Intravenous Trastuzumab for at Least 3 Years | **Compare different  routes of administration** | × Research waste × Nonpublication × Inadequate reporting × Design limitations |
| 65 | NCT01815242 | Adjuvant Dynamic Marker-Adjusted Personalized Therapy Trial Optimizing Risk Assessment and Therapy Response Prediction in Early Breast Cancer - Triple Negative Breast Cancer | **Compare different modalities** | × Research waste × Nonpublication × Inadequate reporting × Design limitations |
| 66 | NCT01816594 | NeoPHOEBE: Neoadjuvant Trastuzumab + BKM120 in Combination With Weekly Paclitaxel in HER2-positive Primary Breast Cancer | **Add therapeutic component** | × Research waste × Nonpublication × Inadequate reporting × Design limitations |
| 67 | NCT01818063 | Carboplatin and Combination Chemotherapy With or Without Veliparib in Treating Patients With Stage IIB-IIIC Breast Cancer | **Add therapeutic component** | √ Research waste √ Nonpublication Inadequate reporting Design limitations |
| 68 | NCT01822314 | Neoadjuvant Chemotherapy With Nab-paclitaxel in Women With HER2-negative High-risk Breast Cancer | **Compare different modalities** | × Research waste × Nonpublication × Inadequate reporting × Design limitations |
| 69 | NCT01835236 | Trastuzumab & Pertuzumab Followed by T-DM1 in MBC | **Remove therapeutic component** | × Research waste × Nonpublication × Inadequate reporting × Design limitations |
| 70 | NCT01856452 | Comparison Study of Sentinel Lymph Node Biopsy by Multimodal Method in Breast Cancer | **Compare different modalities** | √ Research waste × Nonpublication √ Inadequate reporting × Design limitations |
| 71 | NCT01864746 | A Study of Palbociclib in Addition to Standard Endocrine Treatment in Hormone Receptor Positive Her2 Normal Patients With Residual Disease After Neoadjuvant Chemotherapy and Surgery | **Add therapeutic component** | × Research waste × Nonpublication × Inadequate reporting × Design limitations |
| 72 | NCT01875367 | Phase III to Evaluate Patient´s Preference of Subcutaneous Trastuzumab vs Intravenous in HER2+ Advanced Breast Cancer | **Compare different  routes of administration** | √ Research waste × Nonpublication × Inadequate reporting √ Design limitations |
| 73 | NCT01881230 | Evaluate Risk/Benefit of Nab Paclitaxel in Combination With Gemcitabine and Carboplatin Compared to Gemcitabine and Carboplatin in Triple Negative Metastatic Breast Cancer (or Metastatic Triple Negative Breast Cancer) | **Add therapeutic component** | √ Research waste × Nonpublication × Inadequate reporting √ Design limitations |
| 74 | NCT01905592 | A Phase III Trial of Niraparib Versus Physician's Choice in HER2 Negative, Germline BRCA Mutation-positive Breast Cancer Patients | **Compare different modalities** | √ Research waste × Nonpublication √ Inadequate reporting √ Design limitations |
| 75 | NCT01919229 | A Pharmacodynamics Pre-surgical Study of LEE011 in Early Breast Cancer Patients (MONALEESA-1) | **Add therapeutic component** | √ Research waste × Nonpublication √ Inadequate reporting × Design limitations |
| 76 | NCT01923168 | Study of Letrozole With or Without BYL719 or Buparlisib, for the Neoadjuvant Treatment of Postmenopausal Women | **Add therapeutic component** | √ Research waste × Nonpublication × Inadequate reporting √ Design limitations |
| 77 | NCT01928615 | A Study of Subcutaneously Administered Herceptin (Trastuzumab) in Patients With Human Epidermal Growth Factor Receptor 2 (HER2)-Positive Early Breast Cancer | **Compare different  routes of administration** | √ Research waste √ Nonpublication Inadequate reporting Design limitations |
| 78 | NCT01934335 | Effect of Vandetanib on Cellular Markers in Invasive Breast Cancer | **Add therapeutic component** | √ Research waste × Nonpublication √ Inadequate reporting × Design limitations |
| 79 | NCT01941771 | Phase II Trial With Metronomic, Capecitabine Plus Oral Vinorelbine for Metastatic Breast Cancer | **Compare different dose schedules** | √ Research waste × Nonpublication √ Inadequate reporting × Design limitations |
| 80 | NCT01942135 | Palbociclib (PD-0332991) Combined With Fulvestrant In Hormone Receptor+ HER2-Negative Metastatic Breast Cancer After Endocrine Failure (PALOMA-3) | **Add therapeutic component** | × Research waste × Nonpublication × Inadequate reporting × Design limitations |
| 81 | NCT01945775 | A Study Evaluating Talazoparib (BMN 673), a PARP Inhibitor, in Advanced and/or Metastatic Breast Cancer Patients With BRCA Mutation (EMBRACA Study) | **Compare different modalities** | √ Research waste × Nonpublication × Inadequate reporting √ Design limitations |
| 82 | NCT01950182 | Trastuzumab Combined With Chemotherapy or Endocrine Therapy to Treat Metastatic Luminal B2 Breast Cancer | **Remove therapeutic component** | × Research waste × Nonpublication × Inadequate reporting × Design limitations |
| 83 | NCT01953003 | Phase III Study of Vinflunine Plus Capecitabine Versus Capecitabine Alone in Patients With Advanced Breast Cancer | **Add therapeutic component** | √ Research waste √ Nonpublication Inadequate reporting Design limitations |
| 84 | NCT01958021 | Study of Efficacy and Safety of LEE011 in Postmenopausal Women With Advanced Breast Cancer | **Add therapeutic component** | × Research waste × Nonpublication × Inadequate reporting × Design limitations |
| 85 | NCT01966471 | A Study of Trastuzumab Emtansine (Kadcyla) Plus Pertuzumab (Perjeta) Following Anthracyclines in Comparison With Trastuzumab (Herceptin) Plus Pertuzumab and a Taxane Following Anthracyclines as Adjuvant Therapy in Participants With Operable HER2-Positive Primary Breast Cancer | **Compare different modalities** | × Research waste × Nonpublication × Inadequate reporting × Design limitations |
| 86 | NCT01973634 | Comparison of Acute Toxicity and Cost Between Whole Breast Irradiation With Sequential Boost and Simultaneous Integrated Boost After Breast Conserving Surgery. | **Compare different sequences** | √ Research waste × Nonpublication √ Inadequate reporting √ Design limitations |
| 87 | NCT01982448 | Cisplatin vs Paclitaxel for Triple Negative Breast Cancer | **Compare different modalities** | √ Research waste × Nonpublication √ Inadequate reporting √ Design limitations |
| 88 | NCT01989780 | Bevacizumab Plus Paclitaxel Optimization Study With Interventional Aintenance Endocrine Therapy in Breast Cancer | **Compare different sequences** | × Research waste × Nonpublication × Inadequate reporting × Design limitations |
| 89 | NCT01997333 | Study of Glembatumumab Vedotin (CDX-011) in Patients With Metastatic, gpNMB Over-Expressing, Triple Negative Breast Cancer | **Compare different modalities** | √ Research waste × Nonpublication × Inadequate reporting √ Design limitations |
| 90 | NCT02001506 | Neoadjuvant Chemotherapy of 6 Cycles vs 8 Cycles in Node Positve Breast Cancer | **Shorten treatment duration** | × Research waste × Nonpublication × Inadequate reporting × Design limitations |
| 91 | NCT02003209 | Docetaxel, Carboplatin, Trastuzumab, and Pertuzumab With or Without Estrogen Deprivation in Treating Patients With Hormone Receptor-Positive, HER2-Positive Operable or Locally Advanced Breast Cancer | **Add therapeutic component** | √ Research waste √ Nonpublication Inadequate reporting Design limitations |
| 92 | NCT02005887 | TRial on the Endocrine Activity of Neoadjuvant Degarelix | **Compare different modalities** | √ Research waste × Nonpublication × Inadequate reporting √ Design limitations |
| 93 | NCT02007512 | Efficacy and Safety Study of Enzalutamide in Combination With Exemestane in Patients With Advanced Breast Cancer | **Add therapeutic component** | × Research waste × Nonpublication × Inadequate reporting × Design limitations |
| 94 | NCT02008734 | Randomized Phase II Study to Assess PD 0332991 in Breast Cancer | **Add therapeutic component** | √ Research waste × Nonpublication √ Inadequate reporting × Design limitations |
| 95 | NCT02028507 | Phase III Palbociclib With Endocrine Therapy vs. Capecitabine in HR+/HER2- MBC With Resistance to Aromatase Inhibitors | **Compare different modalities** | √ Research waste × Nonpublication × Inadequate reporting √ Design limitations |
| 96 | NCT02032277 | A Study Evaluating Safety and Efficacy of the Addition of ABT-888 Plus Carboplatin Versus the Addition of Carboplatin to Standard Chemotherapy Versus Standard Chemotherapy in Subjects With Early Stage Triple Negative Breast Cancer | **Add therapeutic component** | × Research waste × Nonpublication × Inadequate reporting × Design limitations |
| 97 | NCT02073487 | Neoadjuvant TDM1 With Lapatinib and Abraxane Compared With Trastuzumab Plus Pertuzumab With Paclitaxel | **Compare different modalities** | × Research waste × Nonpublication × Inadequate reporting × Design limitations |
| 98 | NCT02101385 | Randomized Controlled Trial of Genomically Directed Therapy in Patients With Triple Negative Breast Cancer | **Compare different modalities** | √ Research waste × Nonpublication √ Inadequate reporting × Design limitations |
| 99 | NCT02115048 | Clinical Study for the Treatment of Breast Cancer: the Patient Will Receive Afatinib Plus Letrozole or Letrozole Alone | **Add therapeutic component** | √ Research waste √ Nonpublication Inadequate reporting Design limitations |
| 100 | NCT02120417 | A Study of Ruxolitinib in Combination With Capecitabine in Subjects With Advanced or Metastatic HER2-negative Breast Cancer | **Add therapeutic component** | √ Research waste × Nonpublication √ Inadequate reporting × Design limitations |
| 101 | NCT02125344 | A Phase III Trial Comparing Two Dose-dense, Dose-intensified Approaches (ETC and PM(Cb)) for Neoadjuvant Treatment of Patients With High-risk Early Breast Cancer (GeparOcto) | **Compare different modalities** | × Research waste × Nonpublication × Inadequate reporting × Design limitations |
| 102 | NCT02131064 | A Study Evaluating Trastuzumab Emtansine Plus Pertuzumab Compared With Chemotherapy Plus Trastuzumab and Pertuzumab for Participants With Human Epidermal Growth Factor Receptor 2 (HER2)-Positive Breast Cancer | **Compare different modalities** | × Research waste × Nonpublication × Inadequate reporting × Design limitations |
| 103 | NCT02137083 | A Trial Comparing Docetaxel Plus Fulvestrant With Docetaxel in Metastatic Breast Cancer | **Add therapeutic component** | √ Research waste √ Nonpublication Inadequate reporting Design limitations |
| 104 | NCT02144012 | A Study to Evaluate the Efficacy and Safety of Trastuzumab Emtansine Versus the Combination of Trastuzumab Plus Docetaxel in Patients With HER2-positive Breast Cancer | **Compare different modalities** | √ Research waste √ Nonpublication Inadequate reporting Design limitations |
| 105 | NCT02162719 | A Study Assessing the Safety and Efficacy of Adding Ipatasertib to Paclitaxel Treatment in Participants With Breast Cancer That Has Spread Beyond the Initial Site, and the Cancer Does Not Have Certain Hormonal Receptors | **Add therapeutic component** | × Research waste × Nonpublication × Inadequate reporting × Design limitations |
| 106 | NCT02163694 | A Phase 3 Randomized, Placebo-controlled Trial of Carboplatin and Paclitaxel With or Without Veliparib (ABT-888) in HER2-negative Metastatic or Locally Advanced Unresectable BRCA-associated Breast Cancer | **Add therapeutic component** | × Research waste × Nonpublication × Inadequate reporting × Design limitations |
| 107 | NCT02213042 | Evaluation of Biomarkers Associated With Response to Subsequent Therapies in Subjects With HER2-Positive Metastatic Breast Cancer | **Add therapeutic component** | √ Research waste √ Nonpublication Inadequate reporting Design limitations |
| 108 | NCT02213744 | MM-302 Plus Trastuzumab vs. Chemotherapy of Physician's Choice Plus Trastuzumab in HER2-Positive Locally Advanced/Metastatic Breast Cancer Patients | **Compare different modalities** | √ Research waste √ Nonpublication Inadequate reporting Design limitations |
| 109 | NCT02216786 | A Randomized Study of AZD2014 in Combination With Fulvestrant in Metastatic or Advanced Breast Cancer | **Add therapeutic component** | × Research waste × Nonpublication × Inadequate reporting × Design limitations |
| 110 | NCT02225470 | Eribulin Versus Vinorelbine in Subjects With Locally Recurrent or Metastatic Breast Cancer Previously Treated With Anthracyclines and Taxanes | **Compare different modalities** | × Research waste × Nonpublication × Inadequate reporting × Design limitations |
| 111 | NCT02229149 | Phase 2 Study of Standard Chemotherapy With Trastuzumab, Plus or Minus Pertuzumab, for Pre-treated Metastatic Breast Cancer | **Add therapeutic component** | √ Research waste √ Nonpublication Inadequate reporting Design limitations |
| 112 | NCT02253459 | Clinical Study of UTD1 Injection in Combination With Capecitabine in Patients With Advanced and Metastatic Breast Cancer | **Add therapeutic component** | × Research waste × Nonpublication × Inadequate reporting × Design limitations |
| 113 | NCT02263495 | Eribulin Plus Gemcitabine (EG) vs Paclitaxel Plus Gemcitabine (PG) in HER2-Negative Metastatic Breast Cancer | **Compare different modalities** | √ Research waste × Nonpublication × Inadequate reporting √ Design limitations |
| 114 | NCT02273973 | A Study of Neoadjuvant Letrozole + Taselisib Versus Letrozole + Placebo in Post-Menopausal Women With Breast Cancer (LORELEI) | **Add therapeutic component** | × Research waste × Nonpublication × Inadequate reporting × Design limitations |
| 115 | NCT02278120 | Study of Efficacy and Safety in Premenopausal Women With Hormone Receptor Positive, HER2-negative Advanced Breast Cancer | **Add therapeutic component** | × Research waste × Nonpublication × Inadequate reporting × Design limitations |
| 116 | NCT02296801 | A Phase II Randomized Study Evaluating the Biological and Clinical Effects of the Combination of Palbociclib With Letrozole as Neoadjuvant Therapy in Post-Menopausal Women With Estrogen-Receptor Positive Primary Breast Cancer | **Add therapeutic component** | × Research waste × Nonpublication × Inadequate reporting × Design limitations |
| 117 | NCT02297438 | A Study Of Palbociclib (PD-0332991) + Letrozole VS. Placebo+ Letrozole For 1st Line Treatment Of Asian Postmenopausal Women With ER+/HER2- Advanced Breast Cancer [PALOMA-4] | **Add therapeutic component** | × Research waste × Nonpublication × Inadequate reporting × Design limitations |
| 118 | NCT02301988 | A Study of Ipatasertib (GDC-0068) in Combination With Paclitaxel as Neoadjuvant Treatment for Participants With Early Stage Triple Negative Breast Cancer | **Add therapeutic component** | × Research waste × Nonpublication × Inadequate reporting × Design limitations |
| 119 | NCT02322853 | A Multicenter Trial Assessing the Efficacy and Safety of tamOxifen Plus LY2228820 in Advanced or Metastatic Breast Cancer Progressing on aromatasE Inhibitors | **Add therapeutic component** | √ Research waste √ Nonpublication Inadequate reporting Design limitations |
| 120 | NCT02338245 | Study of ASLAN001 in Combination With Capecitabine in MBC That Has Failed on Prior Trastuzumab | **Compare different modalities** | √ Research waste √ Nonpublication Inadequate reporting Design limitations |
| 121 | NCT02340221 | A Study of Taselisib + Fulvestrant Versus Placebo + Fulvestrant in Participants With Advanced or Metastatic Breast Cancer Who Have Disease Recurrence or Progression During or After Aromatase Inhibitor Therapy | **Add therapeutic component** | × Research waste × Nonpublication × Inadequate reporting × Design limitations |
| 122 | NCT02344550 | Study of GnRH-A [Leuprorelin(Lorelin Depot] Plus Leterozole +/- Everolimus for Premenopausal Women With Metastatic Breast Cancer | **Add therapeutic component** | √ Research waste × Nonpublication √ Inadequate reporting × Design limitations |
| 123 | NCT02344940 | Safety of Toremifene and Tamoxifen Therapy in Premenopausal Patients With Operable Breast Cancer | **Compare different modalities** | × Research waste × Nonpublication × Inadequate reporting × Design limitations |
| 124 | NCT02364557 | Testing Whether Treating Breast Cancer Metastases With Surgery or High-Dose Radiation Improves Survival | **Add therapeutic component** | √ Research waste √ Nonpublication Inadequate reporting Design limitations |
| 125 | NCT02365805 | Randomized CT to Evaluate Efficacy of Neoadjuvant Chemotherapy Customized by Levels of BRCA1-HER2 Negative Breast Cancer | **Compare different modalities** | √ Research waste √ Nonpublication Inadequate reporting Design limitations |
| 126 | NCT02370238 | A Double-blind Study of Paclitaxel in Combination With Reparixin or Placebo for Metastatic Triple-Negative Breast Cancer | **Add therapeutic component** | × Research waste × Nonpublication × Inadequate reporting × Design limitations |
| 127 | NCT02374099 | Study to Assess the Efficacy and Safety of the Epigenetic Modifying Effects of CC-486 (Oral Azacitidine) in Combination With Fulvestrant | **Add therapeutic component** | √ Research waste √ Nonpublication Inadequate reporting Design limitations |
| 128 | NCT02375048 | Randomized Study on Postmenopausal Women With Early Stage Breast Cancer: WBI Versus APBI | **Shorten treatment duration** | √ Research waste × Nonpublication √ Inadequate reporting √ Design limitations |
| 129 | NCT02384239 | A Study of Palbociclib in Combination With Fulvestrant or Tamoxifen as Treatment for Metastatic Breast Cancer | **Reduce cumulative dose** | √ Research waste √ Nonpublication Inadequate reporting Design limitations |
| 130 | NCT02387099 | Dose EScalation Induction of EvERolimus | **Reduce cumulative dose** | × Research waste × Nonpublication × Inadequate reporting × Design limitations |
| 131 | NCT02400567 | Efficacy of Letrozole + Palbociclib Combination as Neoadjuvant Treatment of Stage II-IIIA PAM 50 ROR-defined Low or Intermediate Risk Luminal Breast Cancer, in Postmenopausal Women | **Compare different modalities** | × Research waste × Nonpublication × Inadequate reporting × Design limitations |
| 132 | NCT02413320 | Neoadjuvant Study of Two Platinum Regimens in Triple Negative Breast Cancer | **Remove therapeutic component** | × Research waste × Nonpublication × Inadequate reporting × Design limitations |
| 133 | NCT02422615 | Study of Efficacy and Safety of LEE011 in Men and Postmenopausal Women With Advanced Breast Cancer. | **Add therapeutic component** | × Research waste × Nonpublication × Inadequate reporting × Design limitations |
| 134 | NCT02423603 | PAKT: AZD5363 in Combination With Paclitaxel in Triple-Negative Advanced or Metastatic Breast Cancer | **Add therapeutic component** | √ Research waste × Nonpublication √ Inadequate reporting √ Design limitations |
| 135 | NCT02425891 | A Study of Atezolizumab in Combination With Nab-Paclitaxel Compared With Placebo With Nab-Paclitaxel for Participants With Previously Untreated Metastatic Triple-Negative Breast Cancer (IMpassion130) | **Add therapeutic component** | × Research waste × Nonpublication × Inadequate reporting × Design limitations |
| 136 | NCT02435680 | Efficacy Study of MCS110 Given With Carboplatin and Gemcitabine in Advanced Triple Negative Breast Cancer (TNBC) | **Add therapeutic component** | × Research waste × Nonpublication × Inadequate reporting × Design limitations |
| 137 | NCT02437318 | Study Assessing the Efficacy and Safety of Alpelisib Plus Fulvestrant in Men and Postmenopausal Women With Advanced Breast Cancer Which Progressed on or After Aromatase Inhibitor Treatment. | **Add therapeutic component** | × Research waste × Nonpublication × Inadequate reporting × Design limitations |
| 138 | NCT02441946 | A Neoadjuvant Study of Abemaciclib (LY2835219) in Postmenopausal Women With Hormone Receptor Positive, HER2 Negative Breast Cancer | **Add therapeutic component** | √ Research waste × Nonpublication × Inadequate reporting √ Design limitations |
| 139 | NCT02482753 | Trial of Chidamide in Combination With Exemestane in Patients With Advanced Breast Cancer | **Add therapeutic component** | × Research waste × Nonpublication × Inadequate reporting × Design limitations |
| 140 | NCT02491983 | Palbociclib in Combination With Fulvestrant or Letrozole in Patients With ER+, HER2- Advanced Breast Cancer | **Compare different modalities** | × Research waste × Nonpublication × Inadequate reporting × Design limitations |
| 141 | NCT02492711 | Margetuximab Plus Chemotherapy vs Trastuzumab Plus Chemotherapy in the Treatment of HER2+ Metastatic Breast Cancer | **Compare different modalities** | × Research waste × Nonpublication × Inadequate reporting × Design limitations |
| 142 | NCT02511639 | Maintenance Aromatase Inhibitors (AIs)+ Everolimus vs AIs in Hormone Receptor Positive Metastatic Breast Cancer Patients | **Add therapeutic component** | × Research waste × Nonpublication × Inadequate reporting × Design limitations |
| 143 | NCT02514681 | A Phase III Trial of Pertuzumab Retreatment in Previously Pertuzumab Treated Her2-Positive Advanced Breast Cancer | **Add therapeutic component** | × Research waste × Nonpublication × Inadequate reporting × Design limitations |
| 144 | NCT02532400 | Neoadjuvant Aromatase Inhibitor(AI) With Ovarian Suppression Versus Chemotherapy in Premenopausal Breast Cancer Patients | **Remove therapeutic component** | √ Research waste √ Nonpublication Inadequate reporting Design limitations |
| 145 | NCT02546934 | ABX Plus Cisplatin Versus Gemcitabine Plus Cisplatin in Triple Negative Breast Cancer (TNBC) | **Compare different modalities** | × Research waste × Nonpublication × Inadequate reporting × Design limitations |
| 146 | NCT02549430 | To Reverse ENDocrine Resistance Trial - PD 0332991 Monotherapy vs PD 0332991 in Combination With the Endocrine Therapy | **Add therapeutic component** | × Research waste × Nonpublication × Inadequate reporting × Design limitations |
| 147 | NCT02549677 | Epirubicin Versus Docetaxel Plus Cyclophosphamide in Lymph Node Negative, ER-positive, Her2-negative Breast Cancer | **Compare different modalities** | √ Research waste × Nonpublication × Inadequate reporting √ Design limitations |
| 148 | NCT02555657 | Study of Single Agent Pembrolizumab (MK-3475) Versus Single Agent Chemotherapy for Metastatic Triple Negative Breast Cancer (MK-3475-119/KEYNOTE-119) | **Compare different modalities** | × Research waste × Nonpublication × Inadequate reporting × Design limitations |
| 149 | NCT02569801 | A Study of GDC-0810 Versus Fulvestrant in Postmenopausal Women With Advanced or Metastatic Breast Cancer Resistant to Aromatase Inhibitor (AI) Therapy | **Compare different modalities** | √ Research waste √ Nonpublication Inadequate reporting Design limitations |
| 150 | NCT02574455 | Trial of Sacituzumab Govitecan in Participants With Refractory/Relapsed Metastatic Triple-Negative Breast Cancer (TNBC) | **Compare different modalities** | × Research waste × Nonpublication × Inadequate reporting × Design limitations |
| 151 | NCT02585388 | Addition of Aromatase at the Navelbine in Pretreated Metastatic Breast Cancer. | **Add therapeutic component** | × Research waste × Nonpublication × Inadequate reporting × Design limitations |
| 152 | NCT02586025 | Study in Participants With Early-Stage or Locally Advanced Human Epidermal Growth Factor Receptor (HER) 2-Positive Breast Cancer to Evaluate Treatment With Trastuzumab Plus (+) Pertuzumab + Docetaxel Compared With Trastuzumab + Placebo + Docetaxel | **Add therapeutic component** | × Research waste × Nonpublication × Inadequate reporting × Design limitations |
| 153 | NCT02594371 | Ph3 Study To Determine Safety,Tolerability&Tumor Response Of Oraxol Compared To Taxol In Metastatic Breast Cancer | **Compare different  routes of administration** | × Research waste × Nonpublication × Inadequate reporting × Design limitations |
| 154 | NCT02614794 | A Study of Tucatinib vs. Placebo in Combination With Capecitabine & Trastuzumab in Patients With Advanced HER2+ Breast Cancer | **Add therapeutic component** | × Research waste × Nonpublication × Inadequate reporting × Design limitations |
| 155 | NCT02614833 | IMP321 (Eftilagimod Alpha) as Adjunctive to a Standard Chemotherapy Paclitaxel Metastatic Breast Carcinoma | **Add therapeutic component** | × Research waste × Nonpublication × Inadequate reporting × Design limitations |
| 156 | NCT02628613 | Neoadjuvant Paclitaxel Plus Epirubicin Versus Vinorelbine Plus Epirubicin in Breast Cancer With TEKT4 Variations | **Compare different  routes of administration** | × Research waste × Nonpublication × Inadequate reporting × Design limitations |
| 157 | NCT02630693 | Two Different Schedules of Palbociclib + Second Line Endocrine Therapy in Estrogen Receptor Positive, HER2 Neg Advanced/Metastatic Breast Cancer | **Reduce cumulative dose** | × Research waste × Nonpublication × Inadequate reporting × Design limitations |
| 158 | NCT02632045 | Study of Efficacy of Ribociclib After Progression on CDK4/6 Inhibition in Patients With HR+ HER2- Advanced Breast Cancer | **Add therapeutic component** | × Research waste × Nonpublication × Inadequate reporting × Design limitations |
| 159 | NCT02675231 | A Study of Abemaciclib (LY2835219) in Women With HR+, HER2+ Locally Advanced or Metastatic Breast Cancer | **Compare different modalities** | × Research waste × Nonpublication × Inadequate reporting × Design limitations |
| 160 | NCT02685059 | Addition of PD-L1 Antibody MEDI4736 to a Taxane-anthracycline Chemotherapy in Triple Negative Breast Cancer | **Add therapeutic component** | × Research waste × Nonpublication × Inadequate reporting × Design limitations |
| 161 | NCT02730091 | A Open Study of Metronomic Oral Vinorelbine in Combination With Aromatase Inhibitors for the Treatment of Postmenopausal Women With Hormone Receptor Positive,HER2-negative, Advanced Breast Cancer Who Received no Prior Therapy for Advanced Disease | **Add therapeutic component** | √ Research waste √ Nonpublication Inadequate reporting Design limitations |
| 162 | NCT02742051 | A Trial of Neoadjuvant Everolimus Plus Letrozole Versus FEC in Women With ER-positive, HER2-negative Breast Cancer | **Compare different modalities** | √ Research waste × Nonpublication × Inadequate reporting √ Design limitations |
| 163 | NCT02756364 | Sapanisertib in Combination With Fulvestrant in Women With Advanced or Metastatic Breast Cancer After Aromatase Inhibitor Therapy | **Add therapeutic component** | × Research waste × Nonpublication × Inadequate reporting × Design limitations |
| 164 | NCT02771353 | The HeartSpare Plus 1B Trial | **Compare different modalities** | √ Research waste √ Nonpublication Inadequate reporting Design limitations |
| 165 | NCT02783222 | EFficacy and Impact on Function of Two Different Doses of Nab-paclitaxEl in Elderly With advanCed breasT Cancer | **Reduce cumulative dose** | × Research waste × Nonpublication × Inadequate reporting × Design limitations |
| 166 | NCT02783690 | A Trial of 15 Fraction vs 25 Fraction Pencil Beam Scanning Proton Radiotherapy After Mastectomy in Patients Requiring Regional Nodal Irradiation | **Reduce cumulative dose** | × Research waste × Nonpublication × Inadequate reporting × Design limitations |
| 167 | NCT02783794 | BP-C1 in Short-term Treatment of Patients With Metastatic Breast Cancer | **Add therapeutic component** | √ Research waste × Nonpublication √ Inadequate reporting × Design limitations |
| 168 | NCT02789332 | Assessing the Efficacy of Paclitaxel and Olaparib in Comparison to Paclitaxel / Carboplatin Followed by Epirubicin/Cyclophosphamide as Neoadjuvant Chemotherapy in Patients With HER2-negative Early Breast Cancer and Homologous Recombination Deficiency | **Compare different modalities** | × Research waste × Nonpublication × Inadequate reporting × Design limitations |
| 169 | NCT02819518 | Study of Pembrolizumab (MK-3475) Plus Chemotherapy vs. Placebo Plus Chemotherapy for Previously Untreated Locally Recurrent Inoperable or Metastatic Triple Negative Breast Cancer (MK-3475-355/KEYNOTE-355) | **Add therapeutic component** | × Research waste × Nonpublication × Inadequate reporting × Design limitations |
| 170 | NCT02831530 | Randomized Short-term Pre-surgical Study to Assess the Effects of Abemaciclib (LY2835219) in Early Breast Cancer Patients | **Add therapeutic component** | √ Research waste √ Nonpublication Inadequate reporting Design limitations |
| 171 | NCT02866955 | Evaluation of the Efficacy of Estramustine in Patient With Breast Cancer Progression After Treatment With Aromatase Inhibitor. | **Compare different modalities** | √ Research waste √ Nonpublication Inadequate reporting Design limitations |
| 172 | NCT02896855 | A Study to Evaluate the Efficacy and Safety of Pertuzumab + Trastuzumab + Docetaxel Versus Placebo + Trastuzumab + Docetaxel in Previously Untreated Human Epidermal Growth Factor Receptor 2 (HER2)-Positive Metastatic Breast Cancer | **Add therapeutic component** | × Research waste × Nonpublication × Inadequate reporting × Design limitations |
| 173 | NCT02915744 | A Study of Etirinotecan Pegol (NKTR-102) Versus Treatment of Physician's Choice (TPC) in Patients With Metastatic Breast Cancer Who Have Stable Brain Metastases and Have Been Previously Treated With an Anthracycline, a Taxane, and Capecitabine | **Compare different modalities** | × Research waste × Nonpublication × Inadequate reporting × Design limitations |
| 174 | NCT02924883 | A Study to Evaluate the Efficacy and Safety of Trastuzumab Emtansine in Combination With Atezolizumab or Atezolizumab-Placebo in Participants With Human Epidermal Growth Factor-2 (HER2) Positive Locally Advanced or Metastatic Breast Cancer (BC) Who Received Prior Trastuzumab and Taxane Based Therapy | **Add therapeutic component** | × Research waste × Nonpublication × Inadequate reporting × Design limitations |
| 175 | NCT02954055 | MEtronomic TrEatment Option in Advanced bReast cAncer | **Compare different modalities** | × Research waste × Nonpublication × Inadequate reporting × Design limitations |
| 176 | NCT02978495 | Neoadjuvant Carboplatin in Triple Negative Breast Cancer | **Add therapeutic component** | √ Research waste × Nonpublication √ Inadequate reporting × Design limitations |
| 177 | NCT02980965 | Neoadjuvant Chemo-endocrine Therapy Versus Chemotherapy Alone in ER-positive, HER2-negative Breast Cancer | **Add therapeutic component** | × Research waste × Nonpublication × Inadequate reporting × Design limitations |
| 178 | NCT03051659 | A Randomized Phase II Study Of Eribulin Mesylate With or Without Pembrolizumab For Metastatic Hormone Receptor Positive Breast Cancer | **Add therapeutic component** | √ Research waste × Nonpublication √ Inadequate reporting × Design limitations |
| 179 | NCT03078751 | Adjuvant Ribociclib With Endocrine Therapy in Hormone Receptor+/HER2- High Risk Early Breast Cancer | **Add therapeutic component** | √ Research waste √ Nonpublication Inadequate reporting Design limitations |
| 180 | NCT03084939 | Efficacy and Safety of Trastuzumab Emtansine in Chinese Participants With Human Epidermal Growth Factor Receptor 2 (HER2)-Positive Locally Advanced or Metastatic Breast Cancer | **Compare different modalities** | × Research waste × Nonpublication × Inadequate reporting × Design limitations |
| 181 | NCT03125902 | A Study of Atezolizumab and Paclitaxel Versus Placebo and Paclitaxel in Participants With Previously Untreated Locally Advanced or Metastatic Triple Negative Breast Cancer (TNBC) | **Add therapeutic component** | × Research waste × Nonpublication × Inadequate reporting × Design limitations |
| 182 | NCT03140553 | TCH Versus EC-TH as Neoadjuvant Treatment for HER2-Positive Breast Cancer | **Compare different modalities** | × Research waste × Nonpublication × Inadequate reporting × Design limitations |
| 183 | NCT03154749 | DCb (Docetaxel/Carboplatin) Versus EC-D (Epirubicin/Cyclophosphamide Followed by Docetaxe) as Neoadjuvant Chemotherapy for Triple-Negative Breast Cancer | **Compare different modalities** | × Research waste × Nonpublication × Inadequate reporting × Design limitations |
| 184 | NCT03197935 | A Study to Investigate Atezolizumab and Chemotherapy Compared With Placebo and Chemotherapy in the Neoadjuvant Setting in Participants With Early Stage Triple Negative Breast Cancer | **Add therapeutic component** | × Research waste × Nonpublication × Inadequate reporting × Design limitations |
| 185 | NCT03202446 | Randomized Clinical Trial Evaluating the Use of the Laser-Assisted Immunotherapy (LIT/inCVAX) in Advanced Breast Cancer | **Add therapeutic component** | √ Research waste √ Nonpublication Inadequate reporting Design limitations |
| 186 | NCT03206203 | Carboplatin With or Without Atezolizumab in Treating Patients With Stage IV Triple Negative Breast Cancer | **Add therapeutic component** | × Research waste × Nonpublication × Inadequate reporting × Design limitations |
| 187 | NCT03227328 | CDK4/6-inhibitor or Chemotherapy, in Combination with ENDOcrine Therapy, for Advanced Breast Cancer / KENDO | **Compare different modalities** | √ Research waste × Nonpublication × Inadequate reporting √ Design limitations |
| 188 | NCT03241810 | Phase 2 Trial of Seribantumab Plus Fulvestrant in Postmenopausal Women With Metastatic Breast Cancer | **Add therapeutic component** | √ Research waste √ Nonpublication Inadequate reporting Design limitations |
| 189 | NCT03248427 | Neadjuvant Multi-agent Chemotherapy or Letrozole Plus Ribociclib in Luminal B/HER2-negative Breast Cancer. | **Compare different modalities** | × Research waste × Nonpublication × Inadequate reporting × Design limitations |
| 190 | NCT03254654 | Vinorelbine Plus Apatinib Versus Vinorelbine in Advanced Triple-Negative Breast Cancer | **Add therapeutic component** | √ Research waste × Nonpublication √ Inadequate reporting √ Design limitations |
| 191 | NCT03262935 | SYD985 vs. Physician's Choice in Participants With HER2-positive Locally Advanced or Metastatic Breast Cancer | **Compare different modalities** | × Research waste × Nonpublication × Inadequate reporting × Design limitations |
| 192 | NCT03272477 | Study to Compare Neoadjuvant Combination of Trastuzumab and Pertuzumab With Concurrent Taxane Chemotherapy or Endocrine Therapy and Quality of Life Assessment Under Adjuvant Therapy in Operable HER2+/HR+ Breast Cancer Patients | **Remove therapeutic component** | × Research waste × Nonpublication × Inadequate reporting × Design limitations |
| 193 | NCT03289039 | Neratinib +/- Fulvestrant in HER2+, ER+ Metastatic Breast Cancer | **Add therapeutic component** | × Research waste × Nonpublication × Inadequate reporting × Design limitations |
| 194 | NCT03291886 | Phase 2 Study of KHK2375 in Subjects With Advanced or Recurrent Breast Cancer | **Add therapeutic component** | × Research waste × Nonpublication × Inadequate reporting × Design limitations |
| 195 | NCT03306472 | A Pre-operative Window Study of Letrozole Plus PR Agonist (Megestrol Acetate) Versus Letrozole Alone in Post-menopausal Patients With ER-positive Breast Cancer | **Add therapeutic component** | √ Research waste √ Nonpublication Inadequate reporting Design limitations |
| 196 | NCT03312738 | A Study of Everolimus Plus Exemestane in Chinese Postmenopausal Women With Estrogen Receptor Positive, Locally Advanced, Recurrent, or Metastatic Breast Cancer After Recurrence or Progression on Non-steroidal Aromatase Inhibitor | **Add therapeutic component** | × Research waste × Nonpublication × Inadequate reporting × Design limitations |
| 197 | NCT03322215 | HR+/HER2- Advanced Breast Cancer and Endocrine Resistance | **Compare different modalities** | √ Research waste √ Nonpublication Inadequate reporting Design limitations |
| 198 | NCT03326102 | Oral Paclitaxel Efficacy Safety and PK in Recurrent and Metastatic Breast Cancer | **Compare different  routes of administration** | √ Research waste √ Nonpublication Inadequate reporting Design limitations |
| 199 | NCT03326674 | Tesetaxel Plus Reduced Dose of Capecitabine vs. Capecitabine in HER2 Negative, HR Positive, LA/MBC | **Add therapeutic component** | √ Research waste √ Nonpublication Inadequate reporting Design limitations |
| 200 | NCT03329378 | Neoadjuvant Dose-Dense For Early Her2Neu Positive Breast Cancer | **Increase cumulative dose** | √ Research waste √ Nonpublication Inadequate reporting Design limitations |
| 201 | NCT03358004 | the Role of Two Different Metronomic Chemotherapy Regimens in Locally Advanced or Metastatic Triple Negative Breast Cancer Patients (TNBC) as Maintenance Therapy After First Line Treatment | **Add therapeutic component** | √ Research waste √ Nonpublication Inadequate reporting Design limitations |
| 202 | NCT03383679 | Study on Androgen Receptor and Triple Negative Breast Cancer | **Compare different modalities** | × Research waste × Nonpublication × Inadequate reporting × Design limitations |
| 203 | NCT03386162 | SAFIR-PI3K A Phase II Randomized Maintenance Trial Comparing Alpelisib and Fulvestrant Versus Chemotherapy in PIK3CA Mutated Advanced Breast Cancer | **Compare different modalities** | √ Research waste √ Nonpublication Inadequate reporting Design limitations |
| 204 | NCT03409198 | Phase IIb Study Evaluating Immunogenic Chemotherapy Combined with Ipilimumab and Nivolumab in Breast Cancer | **Add therapeutic component** | √ Research waste × Nonpublication × Inadequate reporting √ Design limitations |
| 205 | NCT03447132 | Fulvestrant Versus Fulvestrant Plus Palbociclib in Operable Breast Cancer Responding to Fulvestrant | **Add therapeutic component** | √ Research waste × Nonpublication √ Inadequate reporting √ Design limitations |
| 206 | NCT03462251 | Ribociclib and Endocrine Therapy or Chemotherapy With or Without Bevacizumab for Metastatic Breast Cancer in First Line | **Compare different modalities** | √ Research waste × Nonpublication × Inadequate reporting √ Design limitations |
| 207 | NCT03487666 | OXEL: Immune Checkpoint or Capecitabine or Combination Therapy as Adjuvant Therapy for TNBC With Residual Disease | **Add therapeutic component** | √ Research waste × Nonpublication × Inadequate reporting √ Design limitations |
| 208 | NCT03493854 | A Study to Evaluate the Pharmacokinetics, Efficacy, and Safety of Subcutaneous Administration of the Fixed-Dose Combination of Pertuzumab and Trastuzumab in Combination With Chemotherapy in Participants With HER2-Positive Early Breast Cancer | **Compare different  routes of administration** | × Research waste × Nonpublication × Inadequate reporting × Design limitations |
| 209 | NCT03499899 | A Study of Efficacy and Safety of LAG525 in Combination With Spartalizumab, or With Spartalizumab and Carboplatin, or With Carboplatin, in Patients With Advanced Triple-negative Breast Cancer | **Add therapeutic component** | √ Research waste √ Nonpublication Inadequate reporting Design limitations |
| 210 | NCT03584009 | A Phase II Study Comparing The Efficacy Of Venetoclax + Fulvestrant Vs. Fulvestrant In Women With Estrogen Receptor-Positive, Her2-Negative Locally Advanced Or Metastatic Breast Cancer Who Experienced Disease Recurrence Or Progression During Or After CDK4/6 Inhibitor Therapy | **Add therapeutic component** | √ Research waste × Nonpublication √ Inadequate reporting × Design limitations |
| 211 | NCT03603197 | BP-C1 in Short-term Treatment of Thai Patients With Metastatic Breast Cancer | **Add therapeutic component** | √ Research waste × Nonpublication √ Inadequate reporting × Design limitations |
| 212 | NCT03659136 | The XENERA™ 1 Study Tests Xentuzumab in Combination With Everolimus and Exemestane in Women With Hormone Receptor Positive and HER2-negative Breast Cancer That Has Spread | **Add therapeutic component** | √ Research waste × Nonpublication √ Inadequate reporting × Design limitations |
| 213 | NCT03671330 | Efficacy and Safety of Ribociclib in Pre- and Postmenopausal Chinese Women With HR Positive, HER2-negative, Advanced Breast Cancer. | **Add therapeutic component** | × Research waste × Nonpublication × Inadequate reporting × Design limitations |
| 214 | NCT03674112 | A Study to Evaluate Patient Preference and Satisfaction of Subcutaneous Administration of the Fixed-Dose Combination of Pertuzumab and Trastuzumab in Participants With HER2-Positive Early Breast Cancer | **Compare different  routes of administration** | √ Research waste × Nonpublication √ Inadequate reporting × Design limitations |
| 215 | NCT03674242 | Study of Eryaspase in Combination With Chemotherapy Versus Chemotherapy Alone for the Treatment of TNBC (TRYbeCA-2) | **Add therapeutic component** | √ Research waste √ Nonpublication Inadequate reporting Design limitations |
| 216 | NCT03726879 | A Study To Evaluate the Efficacy and Safety Of Atezolizumab or Placebo in Combination With Neoadjuvant Doxorubicin + Cyclophosphamide Followed By Paclitaxel + Trastuzumab + Pertuzumab In Early Her2-Positive Breast Cancer | **Add therapeutic component** | × Research waste × Nonpublication × Inadequate reporting × Design limitations |
| 217 | NCT03778931 | Phase 3 Trial of Elacestrant Versus Standard of Care for the Treatment of ER+/HER2- Advanced Breast Cancer | **Compare different modalities** | × Research waste × Nonpublication × Inadequate reporting × Design limitations |
| 218 | NCT03786094 | Pivotal Study in HER2 Negative, Locally Recurrent or Metastatic Breast Cancer | **Add therapeutic component** | √ Research waste √ Nonpublication Inadequate reporting Design limitations |
| 219 | NCT03795012 | Trial With Eribulin or Eribulin+ Endocrine Therapy in Locally-recurrent or Metastatic Breast Cancer Patients | **Add therapeutic component** | × Research waste × Nonpublication × Inadequate reporting × Design limitations |
| 220 | NCT03809988 | PALbociclib Rechallenge in horMone Receptor-posItive/HER2- Negative Advanced Breast Cancer (PALMIRA) | **Add therapeutic component** | × Research waste × Nonpublication × Inadequate reporting × Design limitations |
| 221 | NCT03822468 | Study of 2 Ribociclib Doses in Combination With Aromatase Inhibitors in Women With HR+, HER2- Advanced Breast Cancer | **Reduce cumulative dose** | × Research waste × Nonpublication × Inadequate reporting × Design limitations |
| 222 | NCT03839823 | Study to Compare the Combination of Ribociclib Plus Goserelin Acetate With Hormonal Therapy Versus Combination Chemotherapy in Premenopausal or Perimenopausal Patients With Advanced or Metastatic Breast Cancer | **Compare different modalities** | √ Research waste × Nonpublication × Inadequate reporting √ Design limitations |
| 223 | NCT03894007 | Improving Pre-operative Systemic Therapy for Human Epidermal Growth Factor Receptor 2 (HER2) Amplified Breast Cancer | **Add therapeutic component** | √ Research waste √ Nonpublication Inadequate reporting Design limitations |
| 224 | NCT03905343 | Ribociclib-endocrine Combination Therapy Versus Chemotherapy as 1st Line in Visceral mBC | **Compare different modalities** | √ Research waste √ Nonpublication Inadequate reporting Design limitations |
| 225 | NCT03948568 | Evaluating Optimal Timing of Endocrine Therapy and Radiation Therapy in Early-stage Breast Cancer (REaCT-RETT) | **Compare different sequences** | √ Research waste × Nonpublication × Inadequate reporting √ Design limitations |
| 226 | NCT03952325 | Tesetaxel Plus 3 Different PD-(L)1 Inhibitors in Patients With Triple-Negative MBC and Tesetaxel Monotherapy in Patients With HER2-Negative MBC | **Add therapeutic component** | √ Research waste √ Nonpublication Inadequate reporting Design limitations |
| 227 | NCT03969121 | Neoadjuvant Hormonal Therapy Plus Palbociclib in Operable, Hormone Sensitive and HER2-Negative Primary Breast Cancer | **Add therapeutic component** | √ Research waste × Nonpublication √ Inadequate reporting × Design limitations |
| 228 | NCT04031703 | Study Comparing Paclitaxel Plus Carboplatin Versus Anthracyclines Followed by Docetaxel as Adjuvant Chemotherapy for Triple Negative Breast Cancer (PATTERN) | **Compare different modalities** | × Research waste × Nonpublication × Inadequate reporting × Design limitations |
| 229 | NCT04031885 | A Study of Abemaciclib (LY2835219) in Combination With Fulvestrant Compared to Chemotherapy in Women With HR Positive, HER2 Negative Metastatic Breast Cancer | **Compare different modalities** | √ Research waste √ Nonpublication Inadequate reporting Design limitations |
| 230 | NCT04066790 | Pyrotinib or Trastuzumab Plus Nab-paclitaxel as Neoadjuvant Therapy in HER2-positive Breast Cancer | **Compare different modalities** | √ Research waste √ Nonpublication Inadequate reporting Design limitations |
| 231 | NCT04075058 | Hypofractionated Radiation Therapy in Patients With Breast Cancer | **Shorten treatment duration** | √ Research waste × Nonpublication √ Inadequate reporting √ Design limitations |
| 232 | NCT04436744 | A Study to Evaluate the Efficacy, Safety, and Pharmacokinetics of Giredestrant Plus Palbociclib Compared With Anastrozole Plus Palbociclib for Postmenopausal Women With Estrogen Receptor-Positive and HER2-Negative Untreated Early Breast Cancer (coopERA Breast Cancer) | **Compare different modalities** | × Research waste × Nonpublication × Inadequate reporting × Design limitations |
| 233 | NCT04478266 | Amcenestrant (SAR439859) Plus Palbociclib as First Line Therapy for Patients With ER (+) HER2(-) Advanced Breast Cancer | **Compare different modalities** | × Research waste × Nonpublication × Inadequate reporting × Design limitations |
| 234 | NCT04664972 | Comparison of TP and TAC Regimens in Neoadjuvant Treatment of TNBC | **Compare different modalities** | × Research waste × Nonpublication × Inadequate reporting × Design limitations |
| 235 | NCT04676516 | A Phase II Window of Opportunity Trial of PRMT5 Inhibitor, GSK3326595, in Early Stage Breast Cancer | **Add therapeutic component** | √ Research waste √ Nonpublication Inadequate reporting Design limitations |
| 236 | NCT04864405 | Evaluating the Dose Timing (Morning vs Evening) of Endocrine Therapy and Its Effects on Tolerability and Compliance | **Compare different dose schedules** | × Research waste × Nonpublication × Inadequate reporting × Design limitations |
| 237 | NCT04951245 | Ultrasound-assisted CNSs Mapping Versus Dual-tracer-guided Sentinel Lymph Node Biopsy | **Compare different modalities** | × Research waste × Nonpublication × Inadequate reporting × Design limitations |
| 238 | NCT05054751 | GB491 Combined With Fulvestrant for HR+ HER2- Locally Advanced or Metastatic Breast Cancer | **Add therapeutic component** | × Research waste × Nonpublication × Inadequate reporting × Design limitations |
| 239 | NCT05206656 | Safety and Efficacy of Eribullin or Eribulin Combined With Anlotinib in Metastatic Breast Cancer | **Add therapeutic component** | × Research waste × Nonpublication × Inadequate reporting × Design limitations |
| 240 | NCT05591456 | Accelerated Hypofractionated 1 Week Radiotherapy in Breast Cancer Patients | **Shorten treatment duration** | √ Research waste × Nonpublication √ Inadequate reporting × Design limitations |

**Note:** Justifications for classification adopt uniform labels (e.g., “Add therapeutic component” for escalation studies).

Table S3. Reasons for study discontinuation

| **Reasons for discontinuation** | **Overall (N = 45)** | **Discontinuated studies** | | |
| --- | --- | --- | --- | --- |
|  |  | **De-escalation  studies (N = 2)** | **Escalation studies (N = 30)** | **Other treatment optimization studies (N = 13)** |
| Poor recruitment | 18 (40.0) | 2 | 9 | 7 |
| Futility | 7 (15.6) | 0 | 4 | 3 |
| Harm | 5 (11.1) | 0 | 5 | 0 |
| Organizational/strategic reasons | 4 (8.9) | 0 | 2 | 2 |
| Benefit | 0 (0.0) | 0 | 0 | 0 |
| External evidence | 4 (8.9) | 0 | 3 | 1 |
| Limited resources | 8 (17.8) | 0 | 7 | 1 |
| Unspecified reason | 2 (4.4) | 0 | 2 | 0 |
| Percentage of studies discontinued due to a preventable reason | 29/45 (64.4) | 2/2 (100.0) | 18/30 (60.0) | 9/13 (69.2) |
| **Note:** Three studies were discontinued due to multiple reasons. | | | | |

Table S4. Univariate and multivariate analysis for research waste

| **Characteristic** | **Univariate analysis** | |  | **Multivariate analysis** | |
| --- | --- | --- | --- | --- | --- |
|  | **OR (95% CI)** | **P value** |  | **OR (95% CI)** | **P value** |
| Study discontinuation |  | <0.001 |  |  | <0.001 |
| No | Reference |  |  | Reference |  |
| Yes | 8.500 (3.611-20.006) |  |  | 9.227 (3.639-23.398) |  |
| Year of study start |  | 0.989 |  |  |  |
| 2011-2015 | Reference |  |  |  |  |
| 2016-2021 | 1.004 (0.588-1.712) |  |  |  |  |
| Intervention |  | 0.023 |  |  | 0.059 |
| Pharmacological | Reference |  |  |  |  |
| Nonpharmacological | 5.962 (1.278-27.819) |  |  |  |  |
| Phase |  | <0.001 |  |  | 0.387 |
| Phase 2 | Reference |  |  |  |  |
| Phase 2/3, 3 or 4 | 0.382 (0.224-0.653) |  |  |  |  |
| Intent |  | 0.841 |  |  |  |
| Curative | Reference |  |  |  |  |
| Palliative | 1.055 (0.625-1.781) |  |  |  |  |
| Blinding |  | 0.001 |  |  | 0.021 |
| Open-label | Reference |  |  | Reference |  |
| Double-blinded | 0.349 (0.187-0.651) |  |  | 0.399 (0.182-0.872) |  |
| Recruitment |  | 0.197 |  |  | 0.254 |
| National | Reference |  |  |  |  |
| International | 0.715 (0.430-1.189) |  |  |  |  |
| Estimated enrollment |  | <0.001 |  |  | 0.016 |
| Small (≤100) | Reference |  |  | Reference |  |
| Medium (101 to 300) | 0.453 (0.226-0.909) |  |  | 0.526 (0.238-1.162) |  |
| Large (>300) | 0.145 (0.067-0.315) |  |  | 0.192 (0.062-0.596) |  |
| Funding |  | 0.020 |  |  | 0.626 |
| Non-industry | Reference |  |  |  |  |
| Industry | 0.516 (0.295-0.902) |  |  |  |  |
